# An inverse-protein-folding pre-trained model for predicting the mutational effects in protein-protein interactions

**DOI:** 10.1016/j.isci.2026.117693

**Published:** 2026-10-06

**Authors:** Sihong Mao, Siwei Meng, Ye Xiang

**Affiliations:** 1 Beijing Frontier Research Center for Biological Structure, Center for Infection Biology, School of Basic Medical Sciences, Tsinghua University, Beijing 100084, P.R. China; 2 Tsinghua-Peking Joint Center for Life Sciences, Beijing 100084, P.R. China

**Keywords:** protein-protein interaction, protein engineering, mutational effect on affinity, combinatorial mutation effects, transfer learning

## Abstract

Protein-protein interactions (PPIs) are fundamental to vast biological processes. Accurate prediction of mutation-induced changes in PPIs is crucial for protein engineering and drug discovery. Most existing PPI prediction approaches rely on refined physicochemical features or computationally intensive structural sampling. Here, we present delta-G message-passing-neural-network (DGMPNN), a message-passing neural-network framework for predicting the effects of mutations on PPIs. DGMPNN is pre-trained on an inverse protein folding (IPF) task and employs an adaptive residue-level attention pooling mechanism to enable effective transfer learning for predicting mutation-induced changes in protein binding affinity. The model achieves state-of-the-art performance on multiple benchmark datasets and deep mutational scanning (DMS) datasets. Experimental validation using computationally selected multi-site variants of the SARS-CoV-2 RBD-hACE2 complex showed strong agreement between predicted and experimentally measured binding affinities.

## Highlights

• An IPF pre-trained MPNN, DGMPNN, is developed for PPI ΔΔG prediction
• DGMPNN incorporates an SAG-like pooling strategy to enable residue-level interpretability
• DGMPNN achieves SOTA performance on ΔΔG prediction with benchmark datasets
• DGMPNN identifies previously unobserved SARS-CoV2 antibody escaping variants

## Graphical abstract

**Figure:**
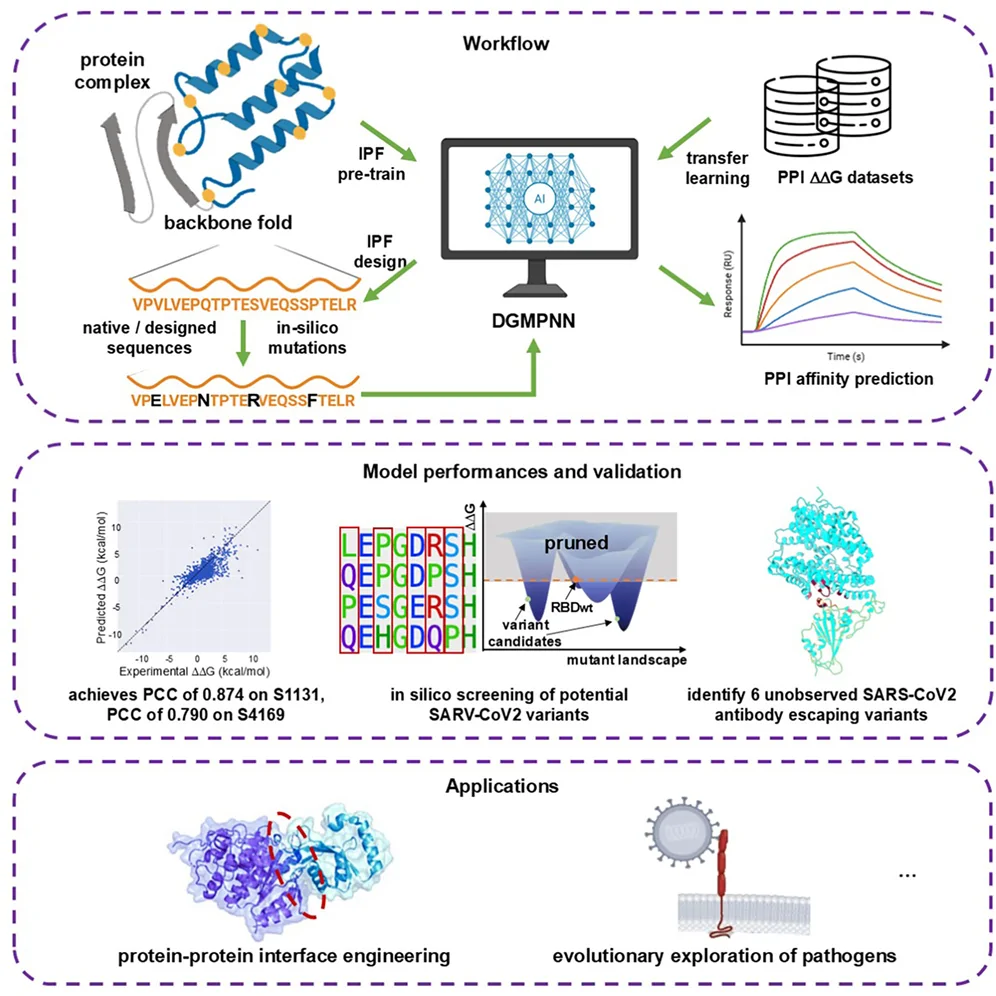

## Teaser

Computational bioinformatics; Protein structure aspects; Structural biology

## Introduction

Cellular physiological functions, such as signal transduction, gene expression regulation, and protein degradation, are primarily mediated through protein-protein interactions (PPIs).^1^^,^^2^ The strength of a PPI is typically evaluated by protein-protein binding affinity, quantified by metrics such as binding free energy (ΔG) or K_D_, which reflect the interaction dynamics. Accurately assessing protein-protein binding affinity is crucial for predicting protein attributes, aiding in the development of bioactive macromolecules.^3^^,^^4^ Mutations, whether arising through natural evolution or intentional design, can significantly impact PPIs, and thereby, alter the attributes of the involved proteins. Predicting the mutation-induced changes in PPIs facilitates the evaluation of the functional consequences of mutations.

Although experimental methods, such as surface plasmon resonance (SPR) and biolayer interferometry (BLI) assays, can accurately assess PPIs, they are often limited by high costs and low throughput.^5^^,^^6^ These limitations restrict their scalability, particularly when evaluating the vast number of protein complexes and their discrete variants found in nature or generated through laboratory engineering.^7^^,^^8^ Over the past two decades, computational methods have been developed to enable rapid and accurate estimation of PPIs and changes in PPI strength. These approaches predict the ΔG of protein complexes, and the change in binding free energy (ΔΔG) between mutated and wild-type (WT) complexes, using physical or statistical energy terms derived from empirical force fields.^9^^,^^10^^,^^11^ Representative methods, such as PoPMuSiC,^12^ Rosetta,^10^ and FoldX,^9^ rely on extensive conformation sampling, and thus, are time consuming and can be inaccurate due to the limitations of discrete conformation sampling.^13^

With the growing size of available PPI data, standard datasets such as the S1131 and S4169 (derived from the Structural database of Kinetics and Energetics of Mutant Protein Interactions (SKEMPI)^14^^,^^15^ were established and have recently been used as the benchmarks in the development of machine-learning-based methods for ΔΔG prediction.^7^^,^^16^^,^^17^^,^^18^ Typical examples of such predictors include mCSM-PPI,^19^ MutaBind,^20^^,^^21^ and BindProfX.^22^ Those ΔΔG predictors took use of the non-linear fitting ability from various machine-learning models.^23^ Comparing with the empirical force-field-based approaches, these machine-learning-based approaches can achieve better accuracy on PPI prediction,^7^ while their accuracy depends on predefined and precalculated input features.

In recent years, a series of deep-learning (DL)-based methods were developed for the prediction of PPI. Among these, PerSpect-EL^24^ and TopNetTree^25^ rely on topological data analysis (TDA), which organizes the topological features of residues as graphs and trains the neural networks to fit the experimental PPI affinity labels. Others, such as GeoPPI,^26^ RDE-Net,^27^ and DiffAffinity^28^ employ self-supervised learning scheme by reconstructing the side-chain conformations. Recent models, such as MpbPPI^29^ and Deep Local Analysis (DLA),^30^ further extend the pre-training framework for PPI predictions. However, both MpbPPI and DLA still need auxiliary features as the model inputs.

DL-based predictors use the pre-training strategy^26^^,^^27^^,^^30^^,^^31^^,^^32^ to leverage abundant unlabeled data and capture the generalizable features that are relevant to PPIs. In theory, these features can be directly transferred to downstream tasks, such as PPI predictions. The protein reverse folding task,^33^^,^^34^ which circumvents the need for explicit side-chain conformation and solely relies on reasonable backbone structures, can be integrated into DL-based predictors for PPI predictions. From the perspective of evaluating the mutation-induced changes in PPIs, methods that use protein reverse folding task as the main pre-training objective can be further explored. Additionally, the recent development and application of deep mutation scanning^35^ (DMS) have significantly expanded the datasets,^36^ which facilitates the further development of PPI prediction methods and models.

In this work, we present a message-passing-neural-network (MPNN) framework for predicting protein-protein binding affinity (K_D_/ΔG) (delta-G MPNN, DGMPNN) and large-scale evaluation of mutation-induced effects across protein-protein interfaces. We applied pre-training on the IPF task for enabling the model to sense the local chemical environment of residues from sequence and structural contexts. Transfer learning is subsequently performed for mutational effect prediction through a residue-level attention-based graph pooling mechanism, which allows the model to gain knowledge from spatial and sequential information on PPI interface. DGMPNN framework achieves state-of-the-art (SOTA) performance without the extra structural sampling and any pre-calculated biochemical features on SKEMPI-derived benchmark datasets and DMS-derived PPI datasets. By efficiently exploring the vast combinatorial sequence space with the model, we identified multisite variants in the SARS-CoV-2 RBD and hACE2 complex with complex epistatic effects that are difficult to detect by using DMS alone. The results showed good agreement between the predictions and experimentally measured data. Our method provides a generalizable platform for accelerating the engineering of protein-based biotechnology and therapeutics, while offering a proactive computational strategy to assess the potential of emerging pathogen variants.

## Results

### Overview of the model framework

We propose a MPNN for accurate and interpretable PPI prediction, namely DGMPNN (Figure 1). DGMPNN gains knowledge of protein structure and PPIfrom IPF pre-training task in an auto-regression scheme, which learns the probability of residue identity on the combined information of protein backbone structure and sequence.^37^^,^^38^ An extra message passing module was then introduced to assign residue-level attention scores. This module functions similarly to a self-attention graph (SAG) pooling block and generates scores conditioned on the unmasked structural and sequence representations produced by the preceding blocks.^39^ The DGMPNN model reweights the residue-level representations with these attention scores and feeds the weighted features into a global pooling block for subsequent prediction of the logarithmic dissociation constant (ln(K_D_)). This predicted K_D_ can then be used for the calculation of ΔΔG.

**Figure 1:**
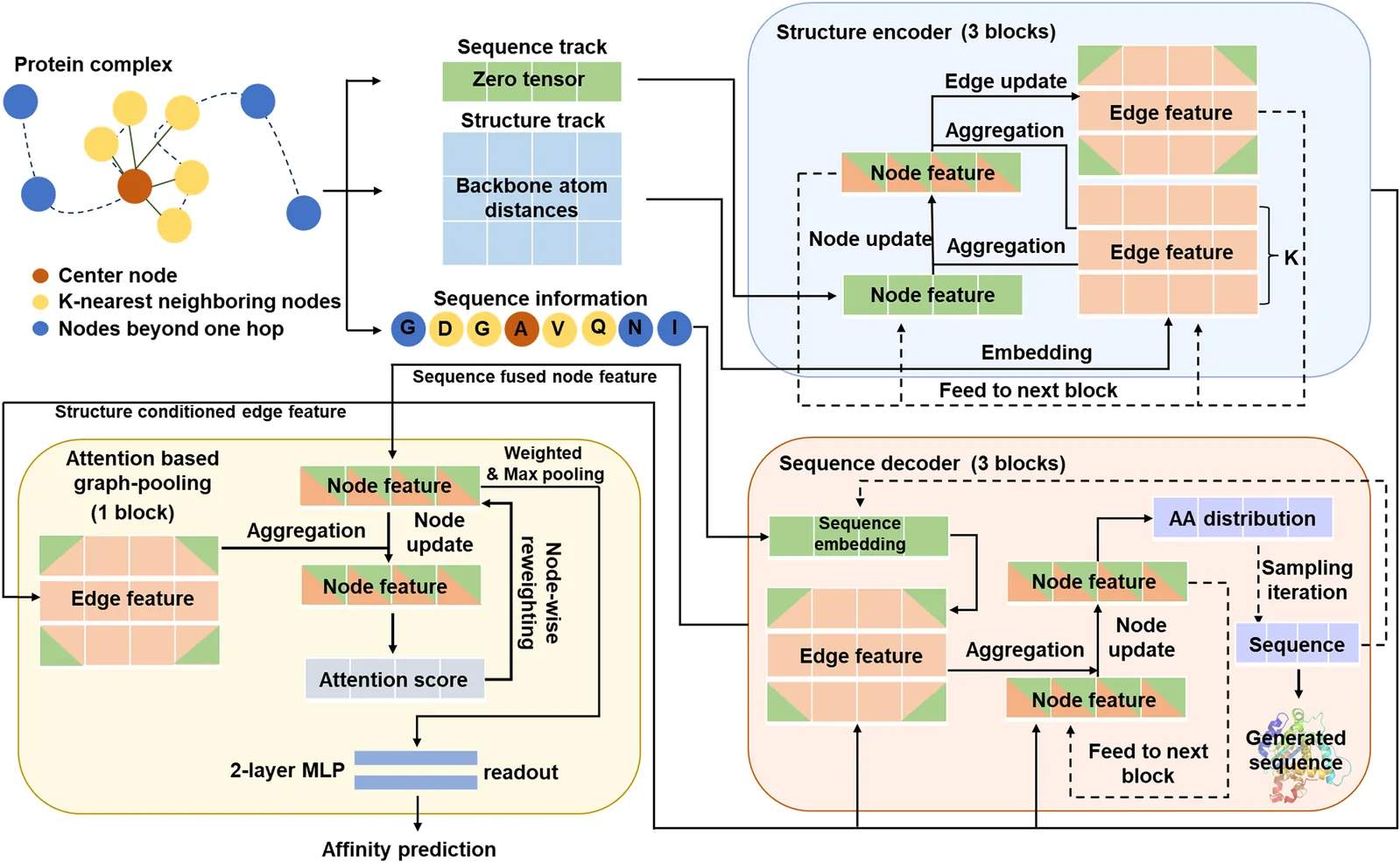
Framework of the DGMPNN model The DGMPNN model integrates structural and sequence information through a graph-based representation, where each node represents an amino acid residue and each edge encodes inter-atomic distances between backbone atoms (25 atomic pairs from backbone atoms Cα, C, N, O, and Cβ) and relative sequence positions along the polypeptide chain(s). In the encoder stage, the model uses zero vectors as the node features to accommodate the backbone-only and sequence-free inputs from the complex structure. Message passing is performed on the k-nearest neighboring nodes (according to the Cα−Cα′ distances and k = 48) and their associated edges along with the center node. Stacked encoder layers expand the receptive field and produce updated node and edge representations enriched with global structural information. In the decoder stage, sequence embeddings are fused into node features with the message passing procedure on the identical k-nearest neighborhood. During the pooling and readout stage, the combined sequence-structure representation undergoes an additional message passing step for node-wise attention reweighting. The final protein representation, modulated by adaptive attention scores, is passed through a 2-layer MLP for down-stream PPI prediction.

For the knowledge-gaining step, we adopted an encoder layer for extracting the spatial information from the protein backbones.^37^ The protein structure was converted to a graph representation. Each node in the graph represents a residue of the protein complex.^40^ The coordinates of the main chain atoms (N, O, Cα, and C) and the side chain Cβ atoms were collected and used to calculate interatomic distances between each two residues. For each protein residue, the distances to the 48 nearest neighboring residues, along with their peptide-chain relationships, were stored in the graph. These 48 nearest neighbors were then defined as the adjacent nodes for each residue. Edge connections were assigned to residues along with their neighbors during the message passing process. Each encoder layer consists of a multilayer perceptron (MLP, 3 layers) with a Gaussian error linear unit (GELU) activation function. Layer normalization on the skip connections, along with the dropout of aggregated messages, was applied at each layer to enhance training performance.^41^ The following decoder layer uses a similar module as that used in the encoder layer, but with the residue information embedded for obtaining the high-dimensional representation of protein structure and its contextual sequence information. The encoder and decoder layers constitute the protein MPNN. In the pre-training stage of the network, the residue identity in the decoder layer was masked. The network was trained by encoding the structural data of a protein or protein complex and eventually recovering the native residue identity at each node in a teaching-force auto-regression way (refer to STAR Methods for details). The pre-trained network was then adapted for PPI affinity prediction with a readout layer inspired by the SAG pooling strategy,^39^^,^^42^ which employs an additional graph neural network (GNN) encoding layer (Figure 1). This extra encoder performs one more round of message passing to generate a multi-head attention score for each node. During this process, node representations from the decoder layer were integrated with edge information from the encoder layer to the multi-head attention scores. Comparing with the traditional SAG pooling strategy, which sets a threshold to discard subgraphs of less significance, all the information on the nodes was kept in the pooling stage of our work frame, thus the message passing process in the pooling stage should be considered as an SAG-like strategy. The global add pooling is introduced to the reweighted feature map as the readout (refer to STAR Methods for details). Then, the readout with adaptive graph attention concatenated with global max pooling is feed into an MLP (2 layers) for the task-specific prediction of PPIs. The mean squared error (MSE) loss between the ln(K_D_) predictions and the experimental labels was used for updating the parameters from the pooling and readout components.

### Selection of training dataset and ablation strategy

We refined the SKEMPI v2.0 dataset by selecting the 2-chain complexes that have a single point mutation and the mutation effect on PPI has been quantified by using experimental assays. The refined data contains 2462 non-redundant mutations and the corresponding 205 unique complexes, designated as S2462. For an unprejudiced comparison of the PPI affinity prediction performance with other approaches, we employ the most widely used S1131 and S4169 for benchmark tests.^43^^,^^44^^,^^45^^,^^46^

We conducted a 10-fold cross validation on these datasets. Our model gave prediction of ln(K_D_) directly. The ΔΔG was further calculated with the predictions from both the variant and the WT(refer to STAR Methods for details). Pearson’s correlation coefficient (PCC) as well as root-mean-square error (RMSE) was calculated as the evaluation metrics.

We performed an ablation study on the SAG-like pooling function to assess the impact of attention head counts and compare our approach against traditional pooling methods. The results showed that the performance on the dataset S2462 was saturated when more than 8 attention heads were used (Table S1). The best performance was obtained by concatenating the SAG-like adaptive pooling module with global max pooling. Compared with traditional max or mean pooling, both the learnable self-attention mechanism and global max pooling contributed to the model’s overall performance gains (Table S1).

### DGMPNN model performance on the benchmark dataset

Our DGMPNN model achieved a PCC of 0.93 and an RMSE of 2.04 on the ln(K_D_) prediction, while achieving a PCC of 0.80 and an RMSE of 1.22 on the ΔΔG prediction, with the dataset S2462 (Figures 2A and 2B). Further analysis of the results showed that the model achieves a better PCC of 0.87 (ΔΔG) on mutations of polar and charged residues, when compared with mutations of hydrophobic residues (PCC = 0.74, ΔΔG) (Figures 2C and 2D). The model also has varied performance on residues located at different positions of the interaction interface. Mutations on residues at the core region of the interface yielded a better PCC of 0.83 (ΔΔG), when compared with those mutations of interface support residues (PCC = 0.66, ΔΔG), interface rim residues (PCC = 0.63, ΔΔG) and non-interface residues (PCC = 0.65, ΔΔG) (Figures 2E–2H).^47^

**Figure 2:**
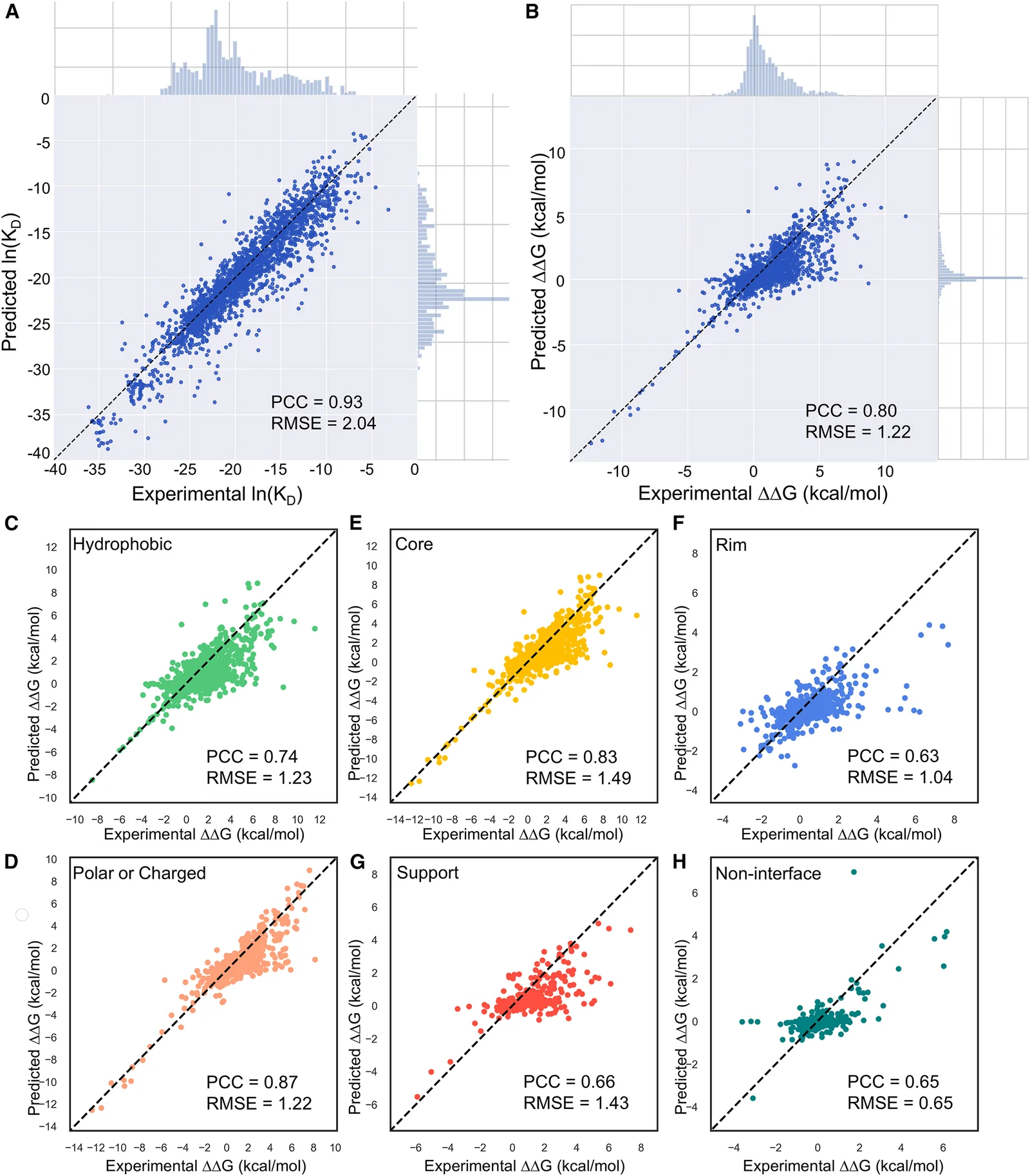
Model performance (A) A scatterplot comparing the logarithmic experimental binding affinity values (ln(*K*_*D*_) values) (*x* axis) and predicted ln(*K*_*D*_) values (*y* axis) for single point mutation variants and their corresponding wild-type two-chain complexes. Histograms aligned to the corresponding axes display the value distributions from the experimental labels and predictions. (B) A scatterplot comparing the experimentally measured ΔΔG and predicted ΔΔG values in kcal/mol. The ΔΔG values are calculated with the equation $\Delta \Delta G=RT\;\ln\left(K_{D_{mut}}∕K_{D_{wt}}\right)$. Histograms aligned to the corresponding axes display the value distributions. (C–H) Scatterplots comparing the measured and predicted ΔΔG values induced by mutations in (C) hydrophobic residues, (D) polar or charged residues, (E) core residues, (F) rim residues, (G) support residues, and (H) non-interface (interior, surface) residues.

With the datasets S1131 and S4169, the two most commonly used benchmarks for PPI affinity prediction task,^19^^,^^24^^,^^26^^,^^29^^,^^31^^,^^48^ we made parallel comparisons of our model with other models that presented a SOTA performance once, including UniBind,^32^ MpbPPI,^29^ Hom-ML-V2,^48^ PerSpect-EL,^24^ GeoPPI,^26^ TopNetTree,^25^ mCSM-PPI2,^19^ MutaBind2,^21^ and BindProfX.^22^ All the models were reported to be trained on the SKEMPI-derived datasets under a 10-fold cross validation scheme. We evaluated DGMPNN with the same 10-fold cross validation scheme. However, the data partitioning details are not available for these reported models. To account for variability arising from data partitioning, the evaluation with DGMPNN was repeated in triplicate, with each test using a different random seed to generate the cross-validation splits (Tables 1 and S2). FoldX^24^ and other energy-based approaches were included as a baseline. The PCC and RMSE metrics of the models were determined under the optimized model architectures and hyper-parameters. Our DGMPNN model achieved an average PCC of 0.875 and 0.790 (on ΔΔG values) on the datasets S1131 and S4169, respectively, demonstrating that the model has a comparable as or even better performance than other models (Table 1).

**Table 1:** Performance landscape of different PPI prediction methods on SKEMPI derived benchmarks

Method	S1131^a^^,^^b^	S4169^a^^,^^b^	References
DGMPNN	**0.874∗**	**0.790**	–
UniBind	**0.870**	0.780	Wang et al.^32^
MpbPPI + FoldX	**0.865**	**0.795**	Yue et al.^29^
MpbPPI + MODELLER	0.847	0.782	Yue et al.^29^
Hom-ML-V2	0.857	**0.800∗**	Liu et al.^48^
PerSpect-EL (M3)	0.853	–	Wee et al.^24^
GeoPPI	0.850	0.780	Liu et al.^26^
TopNetTree	0.760	0.760	Wang et al.^25^
mCSM-PPI2	–	0.760	Rodrigues et al.^19^
MutaBind2	–	0.740	Zhang et al.^21^
BindProfX	0.738	–	Xiong et al.^22^
Profile-score + FoldX	0.738	–	Wee et al.^24^
Profile-score	0.675	–	Szilagyi et al.^49^
FoldX	0.423	0.326	Schymkowitz et al.^9^

a The top 3 PCC values are highlighted in bold, with the highest value further marked with an asterisk.

b The PCC values of DGMPNN are expressed as the mean value across three independent repeats.

To evaluate the model performance under a more challenging scenario, we conducted further tests under a leave-one-complex-out cross-validation scheme using the S1131 dataset. In each test fold, all data belonging to a given protein complex were excluded from training. The final PCC was calculated across all held-out predictions. DGMPNN achieved an overall PCC value of 0.69, which is comparable to SOTA models evaluated under the same benchmark strategy (Table S3).

To investigate whether homologous protein complexes present in the pre-training stage influence downstream predictive performance, we conducted a case study using the protein complex 1R0R from the S1131 dataset. The 1R0R complex consists of two polypeptide chains, a protease and its cognate inhibitor, and is associated with 152 mutational measurements in S1131. The PDB dataset used for pre-training contains 66 homologous complexes of 1R0R, each comprising both the protease and inhibitor chains (the predicted 3D similarity score^50^ is more than 0.8), with sequence identities exceeding 30% for both chains. We removed 1R0R and all its homologous complexes from the pre-training dataset, generating a 1R0R-homolog-excluded PDB dataset. A new DGMPNN model was then pre-trained on this dataset and evaluated using the 1R0R-complex-out cross-validation scheme. Its performance was compared with that of the baseline model pre-trained on the original PDB dataset. On the 1R0R-related data, the 1R0R-homolog-excluded model achieved a PCC of 0.6870, which is comparable to the baseline model that has a PCC of 0.6847 (Table S4). These results suggest that the downstream predictive capability of DGMPNN is not primarily driven by memorization of homologous complexes during pre-training, but instead reflects transferable structural and sequence representations learned from large-scale protein data.

Besides, these data indicate that our model can archive advanced prediction performance in a pure DL way, without using precalculated features, energy-terms, and any additional structural optimization process.

### Model performance on non-standard data

Since the pandemic of COVID-19, abundant high-throughput deep mutation scanning (DMS) data on the interaction of SARS-CoV2 RBD and hACE2 were accumulated.^51^^,^^52^^,^^53^^,^^54^^,^^55^^,^^56^ These DMS data can be converted into the apparent K_D_ values as well as the ΔΔG values. We selected 23 single point mutations from the DMS data, which have also been measured by using the experimental assays with SPR. The DMS-derived ΔΔG values have a PCC of 0.97 with those ΔΔG values derived from SPR measurements, indicating a good consistence between data obtained from different methods (Table 2). The converted DMS data provide excellent resources of non-standard data for further validation of our model as well as for evaluating the capability of our model in gaining knowledge from learning.^51^^,^^52^^,^^53^^,^^54^^,^^55^ There are only four SARS-CoV RBD and hACE2 related data points in our training dataset S2462, while none of the SARS-CoV2 RBD related data were included. Our model (without any DMS data incorporated in training, denoted as “woDMS”) prediction achieved a PCC of 0.45 (0.33 when the predicted ΔΔG values rounded to 2 decimal places) with the SPR-derived ΔΔG and a PCC of 0.47 (0.35 when the predicted ΔΔG values rounded to 2 decimal places) with the DMS-derived ΔΔG on the collected mutants of SARS-CoV2 RBD-hACE2. Our model predictions report a PCC of 0.63 (0.63 when the predicted ΔΔG values rounded to 2 decimal places) if the SARS-CoV2 RBD DMS data were incorporated in training (denoted as “wDMS”). Our model archives numerically higher PCC than other reported models did, which yielded PCCs from 0.20 to 0.31 (Table 2). The PCC value is comparable to that reported by Rosetta flex ΔΔG, which used structure resampling and empirical potential energy function for prediction and achieved a PCC value of 0.36 against the SPR-derived ΔΔG values. However, the PCC value of our model is worse than that obtained by free energy perturbation (FEP) approach, which can achieve a PCC of 0.59 against the SPR-derived ΔΔG (Table 2).

**Table 2:** Performance comparison of different approaches on the SARS-CoV-2 sub dataset

Mutation^a^		ΔΔG SPR	ΔΔG DMS	ΔΔG DGMPNN (woDMS)^c^	ΔΔG DGMPNN (wDMS)^d^	ΔΔG FEP	ΔΔG Mutabind2	ΔΔG mCSM-PPI2^d^	ΔΔG SAAMBE-3D^d^	ΔΔG BeAtMusic^d^	Rosetta flex ΔΔG
N501Y		−0.8	−1.5	-0.2 (−0.20)	−2.7 (−2.71)	−1.2	0.7	0.5	0.0	0.1	0.4
Y453F		−0.7	−1.9	0.0 (0.02)	0.0 (-0.01)	−0.6	−0.2	0.1	0.1	0.3	−0.2
S477N		−0.5	−0.8	0.0 (0.04)	0.0 (0.00)	−0.1	−0.1	−0.1	0.2	0.1	0.1
N501T		−0.5	−1.2	-0.1 (−0.10)	−0.7 (−0.73)	−1.8	−0.6	-0.9	−0.1	0.4	−0.4
N439K		−0.1	−0.3	0.1 (0.05)	0.2 (0.19)	0.6	−0.1	0.5	0.6	0.3	0.0
N440K		0.0	−0.3	0.0 (0.02)	0.0 (0.00)	−0.4	0.1	−0.1	0.5	0.0	0.0
F490S		0.0	−0.1	-0.1 (−0.06)	0.0 (-0.01)	−0.1	0.6	0.2	0.8	0.7	0.0
L452M		0.0	−0.2	0.0 (0.03)	0.0 (0.00)	0.2	0.0	0.5	−0.3	0.2	0.0
L452R		0.0	−0.2	0.0 (−0.01)	0.0 (0.01)	−0.3	−0.8	0.0	0.1	0.2	0.0
E484Q		0.1	0.0	0.0 (−0.01)	0.0 (0.00)	0.3	0.1	0.3	0.5	0.2	0.0
T478K		0.1	0.2	0.0 (0.04)	0.0 (0.00)	0.0	0.2	0.0	0.1	0.2	0.1
N481K		0.1	0.0	0.0 (0.02)	0.0 (0.00)	−0.1	0.0	0.0	0.6	0.2	0.0
E484K		0.1	−0.1	0.0 (0.03)	0.0 (0.00)	0.2	0.2	0.3	0.3	0.1	−0.1
Q498R		0.2	0.5	0.2 (0.25)	−0.2 (−0.20)	2.7	0.4	1.4	1.1	0.8	−0.6
S477I		0.2	0.3	0.1 (0.05)	0.7 (0.68)	0.0	0.1	0.0	0.0	0.2	0.0
G446V		0.2	0.7	0.2 (0.16)	0.0 (−0.02)	0.4	−0.7	0.1	−0.3	1.3	0.0
T478R		0.2	0.0	0.0 (0.03)	0.0 (0.00)	−0.1	0.1	0.0	0.3	0.2	0.0
S477R		0.3	0.1	0.1 (0.06)	0.5 (0.49)	−0.2	0.2	−0.1	0.0	0.1	-0.2
A475V		0.3	0.6	0.0 (−0.03)	0.9 (0.90)	−1.1	0.9	0.0	0.1	0.1	0.2
L455F		0.4	0.8	0.0 (0.00)	0.0 (0.00)	1.7	1.5	−0.7	0.2	-0.1	−0.6
K417T		0.4	0.7	0.0 (−0.04)	0.0 (0.00)	0.9	0.2	0.4	0.5	0.5	0.7
F486L		0.6	1.0	0.0 (−0.03)	0.6 (0.58)	1.3	0.3	0.9	0.5	0.7	1.8
K417N		0.6	1.2	0.0 (−0.02)	0.0 (0.01)	0.9	0.6	0.5	0.4	0.5	0.9
vs SPR^b^	PCC	–	0.97	**0.45** (0.33)	**0.63∗** (0.63)	**0.59**	0.31	0.20	0.24	0.22	0.36
	RMSE	–	0.43	**0.33∗** (0.35)	0.51 (0.51)	0.79	0.52	0.53	**0.47**	**0.49**	0.49
vs DMS^b^	PCC	0.97	–	**0.47** (0.35)	**0.54** (0.55)	**0.65∗**	0.34	0.25	0.24	0.31	0.35
	RMSE	0.43	–	**0.72** (0.73)	**0.68∗** (0.68)	**0.75**	0.77	0.80	0.80	0.79	0.75

a Single point mutations are based on the wild-type RBD context and the ΔΔG are listed in kcal/mol.

b PCC and RMSE were calculated against the experimental SPR and DMS labels, respectively. The top 3 values are highlighted in bold, and the highest value further denoted by an asterisk.

c DMS data were not incorporated in training, denoted as woDMS.

d DMS data were incorporated in training, denoted as wDMS.

We further validated the performance of the wDMS model using a dataset of the *Clostridium thermocellum* complex dockerin-cohesin from the MaveDB database. The wDMS model achieved a PCC of 0.514 and 0.242 on the interface residues and all residues, respectively, for the ΔΔG prediction, which outperformed the corresponding FoldX prediction values (0.460 and 0.145, respectively). Of note, the woDMS model has a worse performance on this specific case, with the PCCs of 0.396 and 0.133, respectively (Table S5). This discrepancy may reflect the systematic bias between these distinct measurement techniques. Fine-tuning on the same type of data can enhance the model’s capacity.

### The gaining knowledge ability of DGMPNN

From the SAG-like pooling components of our model, we can obtain residue-specific attention scores to evaluate the contribution of each node (residue) to the PPIs. This feature provides a distinct advantage of the model, through which key residues that have significant contribution to the PPIs could be identified. We constructed a heatmap (Figure S1) with min-max normalized attention scores for residues from both the RBD and hACE2 in the complex. The complex structure of RBD and hACE2 is colored according to the head-averaged attention values (Figure 3). Analysis of the heatmap and the complex structure showed that residues Q493, Q498, T500, N501, and Y505 of RBD, as well as residues H34, D38, Y41, K353, and D355 of hACE2, are identified as having key contributions to the PPI (Figure 3). These residues are well-established as critical to the RBD-hACE2 interaction and their role has been extensively verified by previous studies,^57^^,^^58^^,^^59^ indicating our model’s ability to distinguish the key contributors to PPIs.

**Figure 3:**
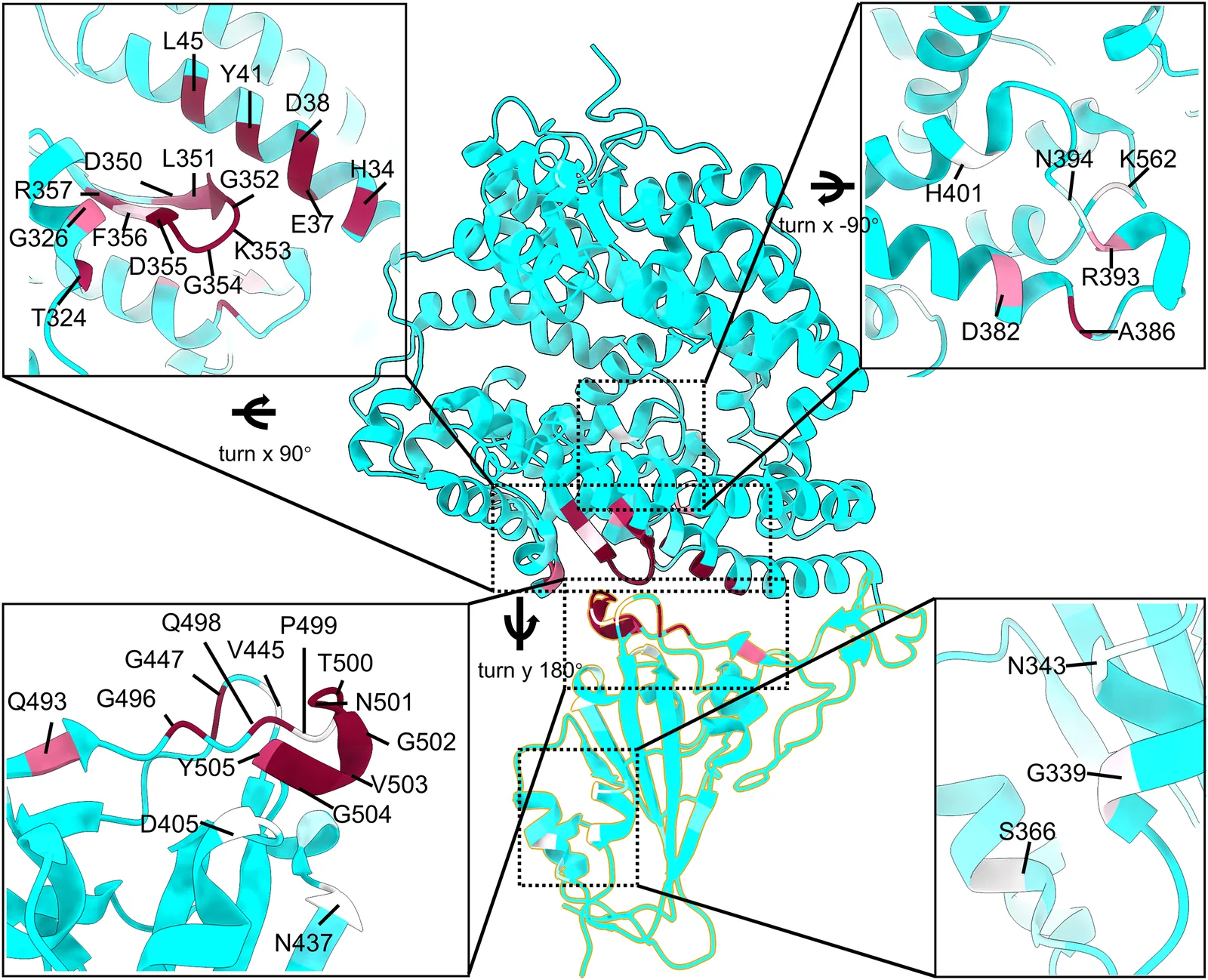
Visualization of the residue-level attention scores of the hACE2 and SARS-CoV2 RBD complex The structure of the hACE2-SARS-CoV2 RBD complex (PDB: 6M0J) is shown in ribbon representation, with the RBD structure outlined by orange lines. Residues of the complex are color-coded from cyan to deep brown according to the head-averaged attention scores. Insets showing zoomed-in views around the key interaction hotspots.

It is worth mentioning that several residues, including G339, N343, S366, and G447, that are not directly involved in the ACE2-binding interface also have significant attention assignments (Figure 3), though no relevant data point to these residue sites exists in the training data. Further literature searches showed that the roles of those non-interface residues affect ACE2 binding through different mechanisms.^60^^,^^61^^,^^62^^,^^63^ Residues G339 and N343 participate in the secondary structure rearrangement of RBD,^60^ which is required for binding ACE2. The glycan attached to residue N343 was shown to interact with the glycans attached to residue N322 of ACE2.^60^^,^^61^^,^^63^ Residue S366 was shown to have significant movement in molecular dynamics (MD) simulation of WT RBD and contributes to the RBD structure rearrangement while binding ACE2^60^^,^^61^; Residue G447 was shown to affect ACE2 binding by destabilizing RBD^63^ or participating in the interaction with heparan sulfate proteoglycan (HSPG) which facilitates the RBD conformational transition to the “up” state.^62^^,^^64^^,^^65^ These data indicate that our model is capable of gaining generalizable knowledge of PPIs from the training data.

Analysis of the internal relationship among the attention scores, we observed correlated changes of the attention scores on some mutated residues and their neighboring residues. These include residues Q493, N501, and Q498 of RBD. The mutant Q493R causes significant changes of the attention scores on neighboring residues L455 of RBD and K31 of ACE2, also mild change on F490 of RBD and H34 and E35 of ACE2 (Figures 4A and 4B). Further structural comparisons of the WT RBD-ACE2 complex and the mutant RBD-ACE2 complex showed that the mutation Q493R disrupts the hydrogen bond between RBD-Q493 and ACE2-K31. However, ACE2-K31 re-establishes a hydrogen bond with Y489 of RBD. Thus, these changes in the hydrogen bonds mediated interactions and repacking of sidechains in local environment cause significant changes of the attention scores on residues ACE2-K31 and RBD-L455 (Figures 4A and 4B). Similarly, mutations in residues N501 and Q498 cause the disruption and establishment of interactions on neighboring residues, resulting in the subtle change of attention scores on neighboring residues (Figures 4C–4F). The accompanying change of the attention scores reveals the combinatorial and epistatic effects of local correlated mutations. These results, therefore, indicate that the multisite-mutations should be considered in PPI analysis rather than only considering the change of a single residue.

**Figure 4:**
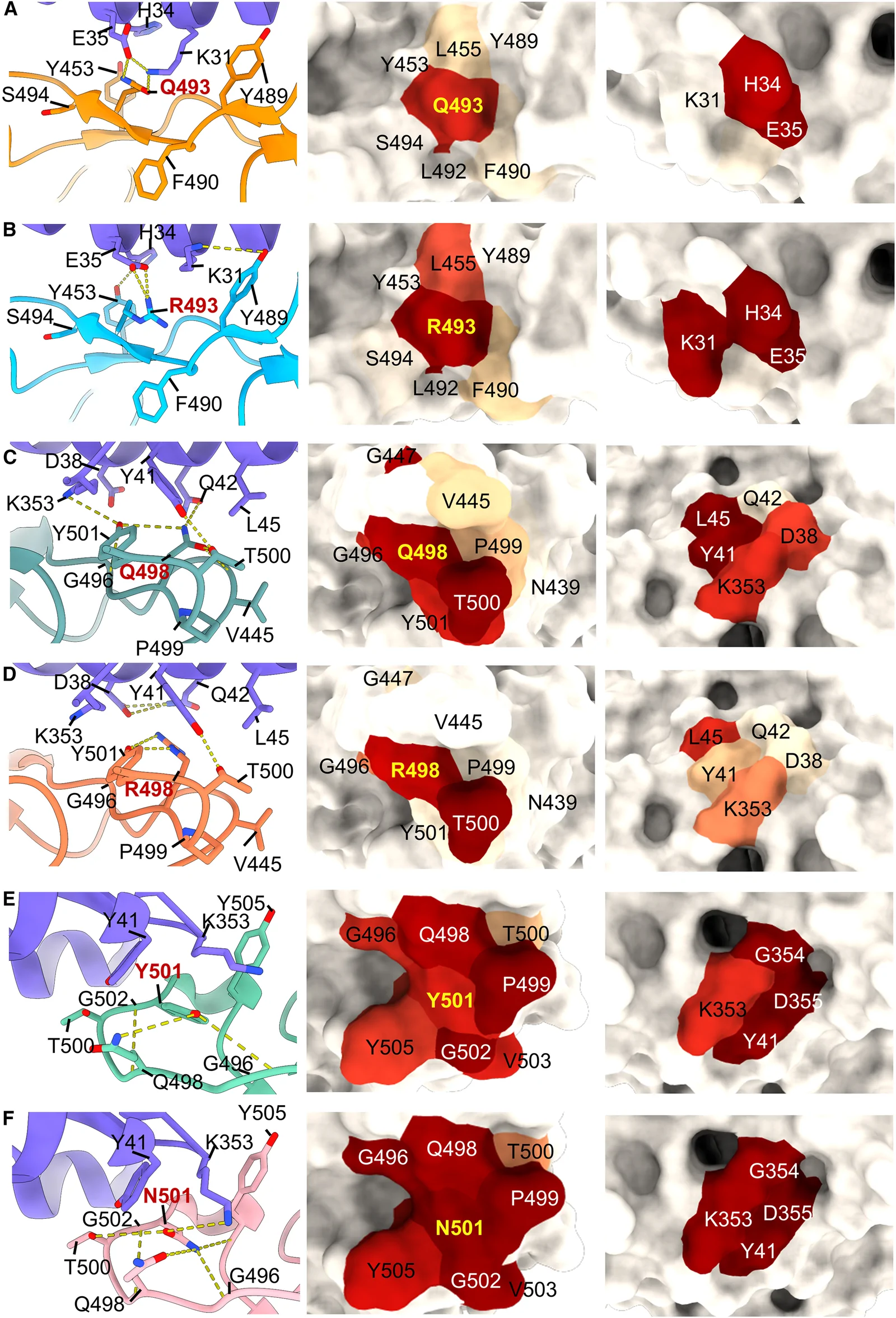
Correlated changes of the attention scores around the key interaction positions (A and B) Left: ribbon and stick representations showing the local region surrounding residue Q493/R493. Dash lines indicate hydrogen bonds. Middle and right: surface-rendered representations showing the local region surrounding Q493/R493 of RBD and the corresponding contact region on hACE2. Surfaces are colored according to head-averaged attention scores. (C and D) Left: ribbon and stick representations showing the local region surrounding residue Q498/R498. Dash lines indicate hydrogen bonds. Middle and right: surface-rendered representations showing the local region surrounding Q498/R498 of RBD and the corresponding contact region on hACE2. Surfaces are colored according to head-averaged attention scores. (E and F) Left: ribbon and stick representations showing the local region surrounding residue N501/Y501. Dash lines indicate hydrogen bonds. Middle and right: surface-rendered representations showing the local region surrounding N501/Y501 of RBD and the corresponding contact region on hACE2. Surfaces are colored according to head-averaged attention scores.

To quantitatively evaluate the relationship between attention scores and mutation-induced ΔΔG, we calculated the correlation between the residue-level attention scores and experimentally measured ΔΔG values. Across the entire RBD, the correlation between the mean attention score and the mean ΔΔG was relatively weak (Pearson’s r = 0.140, Spearman’s ρ = 0.221). However, stratified analysis revealed significant regional differences. Within the receptor binding motif (RBM) region, the correlation improves notably (Pearson’s r = 0.189, Spearman’s ρ = 0.508), whereas it was essentially absent in the non-RBM regions (Table S6). This clear numerical contrast supports the feasibility of using attention weights to interpret residue-level contributions to PPIs. The modest global correlation across the entire RBD suggests an inherent limitation of experimental ΔΔG measurements, which reflects not only changes in direct binding interactions, but also indirect, coupled effects arising from alterations in other physicochemical properties.

### Predictive indications of SARS-CoV-2 evolution by DGMPNN

We further trained our model with the converted DMS data from SARS-COV-2 BA.1 strain. Under a 10-fold cross-validation scheme, the model achieved a PCC of 0.92 on the converted DMS-derived ΔΔG of the SARS-COV-2 RBD and hACE2 (Figure 5A). Due to the lack of SPR or BLI data for the BA.1 strain, we conducted an independent test using an external dataset consisting of 33 WT-based RBD mutants (including 27 single-site variants and 6 multi-site variants). The K_D_ values of these mutants with hACE2 were experimentally measured by using SPR or BLI in previous studies.^43^^,^^44^^,^^45^ Our model achieved a PCC of 0.82 and an RMSE of 0.89 on the 33 mutants against their experimental ΔΔG values (Figure 5B). The multisite-mutations in a local region should be considered to explore the effect of the mutations on viral evolution under the pressure of natural selection, such as the pressure from neutralization antibodies. The observation of correlated changes in local regions suggests that the model is capable of sensing and capturing the combined effects brought by single mutation sites (Figure 4). This feature can be used for evaluating mutants bearing multi-site mutations. We collected data on different SARS-CoV-2 variants, each containing multi-site mutations at the interaction interface with hACE2 (Table S7). The predicted ΔΔG values showed a strong correlation with experimentally determined values (PCC = 0.57), while the direct summation of ΔΔG across those single-point mutations showed a weak correlation (PCC = 0.09) (Table S7). The result suggests the existence of synergistic and epistatic effects regarding multi-site mutations, which supports the model’s capability to assess the non-linear impact of multi-site mutations.

**Figure 5:**
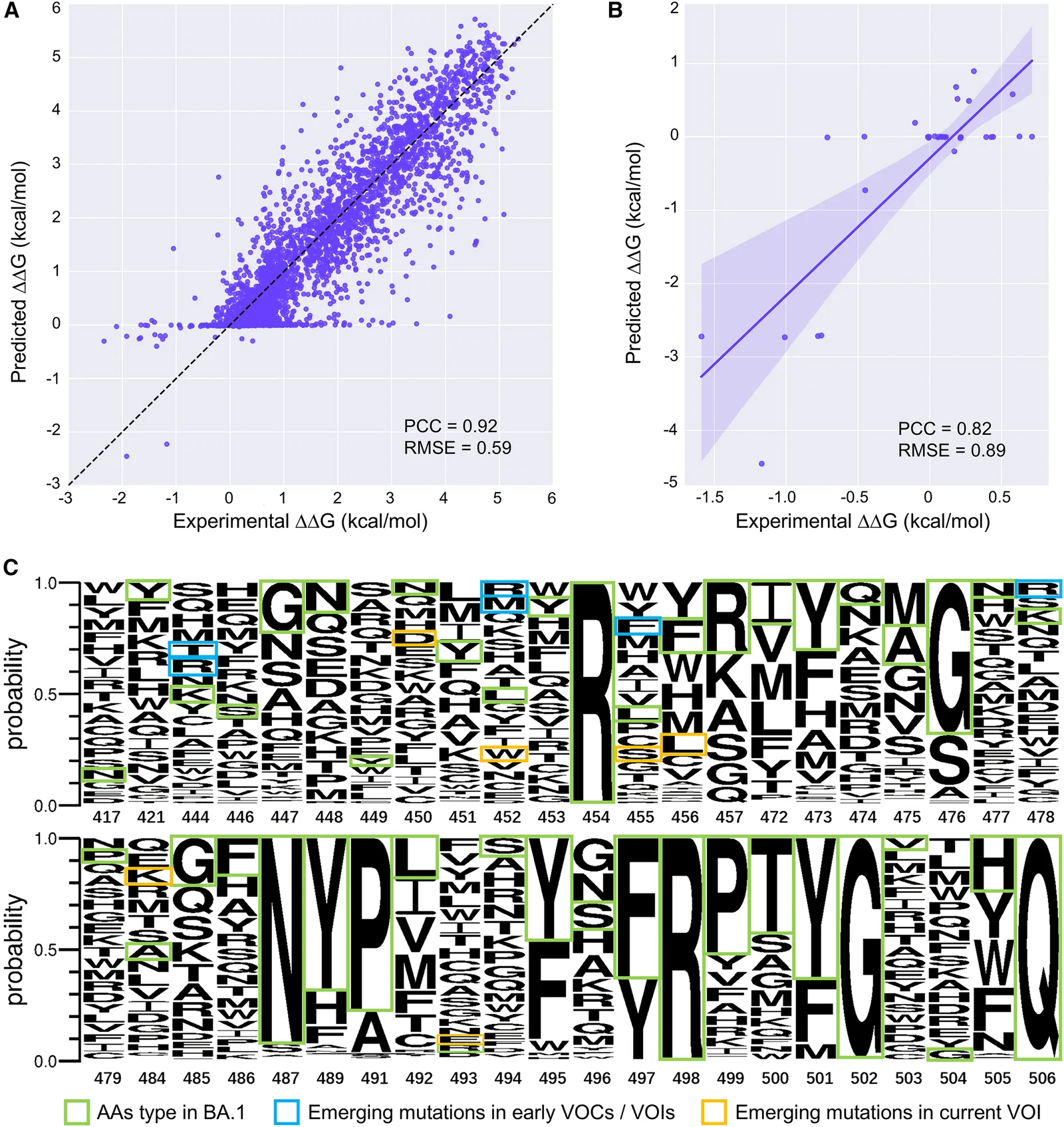
Model performance on DMS data and site preference revealed by *in silico* patch-based multi-site mutation screening (A) Scatterplot comparing DGMPNN predictions with experimental ΔΔG values derived from the DMS dataset. The *x* axis represents experimental ΔΔG values from the DMS dataset, and the *y* axis represents predicted ΔΔG values from the model (in kcal/mol). (B) Scatterplot comparing DGMPNN predictions with an external experimental binding affinity dataset of hACE2 and RBD mutants (from SPR and BLI assays). The *x* axis represents the experimental ΔΔG values, and the *y* axis represents predicted ΔΔG values from the model (in kcal/mol). (C) Sequence logo (SeqLogo) showing standardized amino acid distribution across 44 different sites on the RBD. Each column represents an RBD residue (site index shown at the bottom). The height of each 1-letter amino acid symbol reflects the probability of that residue. The letters were sorted in descending order of frequency. The native amino acid identities from BA.1 strain at the site are marked in green box. Those emerging mutations from previous VOCs/VOIs or current VOI are highlighted in green box and orange box respectively.

We further performed a systematic *in silico* exploration to evaluate multi-site mutations of the RBD by using the DMS data trained model (on SARS-CoV2 BA.1 strain). To reduce the computation time, we divided the RBM region into small patches, each of which contains five residue sites. A total of 21 patches were generated to cover the RBM region (Table S8). Extensive exploration of 3,200,000 (20^5^) possible combinations was performed on each patch. Mutants that can still maintain the binding affinity with the receptor (ΔG_MUT_ <= ΔG_WT_) were selected. These mutants were then analyzed to identify possible mutations that can facilitate the escape of virus from various neutralizing antibodies that were already characterized in previous literatures.^52^^,^^53^

The results depicted in the SeqLogo^66^ graph (Figure 5C) showed that raw identities of BA.1 RBD on ∼66% (29/44) sites are strongly favored by receptor binding. Some of these residue sites have single dominant amino acid identity, such as R454, G476, N487, P491, F497, R498 (Q498R from WT), P499, Y501 (N501Y from WT), G502, and Q506. Those residues are highly conserved and barely replaced by other amino acids, which is consistent with the result of current natural evolution of the virus.^67^^,^^68^^,^^69^ We also noticed that some BA.1 mutations are statistically preferred in calculation and well-kept in different variants of SARS-CoV-2,^70^^,^^71^ including S477N, T478K, Y505H. Some mutations, which were observed in series of earlier circulating variants of concern (VOCs) or variants of interest (VOIs), including K444T (BQ.1), K444R (BA.2.3.20), Y449H (C.1.2), L452M (BA.2.3.20), L452R (BA.4/BA.5), L455F (XBB.1.5), and K478R (XBB.1.16), are also enriched in our prediction (comparing to the raw identities in BA.1). Other well-known mutations in present VOIs, including N450D (BA.2.86), L452W (BA.2.86), A484K (BA.2.86), L455S (JN.1), and F456L (KP.3), show lower but comparable frequency to the identities at the positions of BA.1. The reduced dependence of recent high-fitness variants on mutations that enhance receptor binding affinity suggests that shifted selective forces may engage in shaping the SARS-CoV-2 evolution (Figure 5C). Notably, several single point mutations that have the strongest receptor binding affinity in the DMS dataset were not the most enriched in the patch-based analysis. These include sites 417 (N417F), 446 (S446I), 449 (Y449N), 453 (Y453F), 475 (S475A), 478 (K478S), and 504 (G504G) of the RBD, suggesting that epistasis and synergistic effects in multi-site mutants can significantly alter the contribution of individual mutations.

### Validation of the multisite mutation predictions using RBD-hACE2

We selected at each site residues of high frequency based on the SeqLogo^66^ graph generated from the patch-based analysis. The selection was further refined by considering the nucleotide accessibility and the antibody escape ability. Approximately 31 million variants were generated by the combinations of different residues in the selection (Table S9). These variants were then assessed by our model and more than 4.4 million variants maintaining a comparable ΔG as that of the WTRBD were obtained to constitute the library “ESCAPE”. None of these mutation combinations have occurred in the current variants of SARS-CoV-2, suggesting a vast unexplored mutagenesis space of SARS-CoV-2 RBD.

We randomly picked 9 BA.1 RBD variants from the library with varied number of mutations (from 4 to 14) and varied range of predicted ACE2 binding affinities (Table S10), namely RBD-4M (Y449D, L452R, A484K, and L455W), RBD-5M-A (N450T, K444E, A484K, L455W, and F486S), RBD-5M-B (V503D, Y449D, S446V, L452R, and A484K), RBD-6M (S446V, K444E, L452R, A484K, L455W, and F486S), RBD-7M (L492V, Y495F, V503D, N450K, L452R, A484K, and L455W), RBD-8M-A (S446V, N450T, K444E, L452R, A484K, L455W, F486S, and N448K), RBD-8M-B (L492V, V503D, Y449D, R493K, N450K, L452R, A484K, and L455W), RBD-10M (L492V, Y495F, V503D, Y449D, R493K, N450K, L452R, A484K, L455W, and Q474E), and RBD-14M (L492V, S496R, N417E, Y449D, S446V, R493K, K444E, L452R, A484K, Y453H, G504D, L455W, Y451F, and Q474E) (Figure 6). We produced the recombinant proteins and used SEC to purify and analyze the proteins (Figures S2 and S3). Then we experimentally verified their receptor binding affinities as well as their binding to a panel of 4 broadly neutralizing antibodies BD30-614, LY-CoV1404 (bebtelovimab), BD55-3372, and BD55-5514 (SA55) (Figures 7A, 7B, S4, and S5). Among the selected broadly neutralizing antibodies, BD30-614, LY-CoV1404, and BD55-3372 are reported to neutralize WT, BA.1, BA.2, BA.2.12, and BA.4/BA.5 efficiently, while BD55-5514 can further neutralize BA2.86, XBB.1.5, and JN.1 strains.^54^^,^^55^^,^^72^ Based on our prediction, these mutants could maintain the binding affinity as that of the WT RBD. BLI data showed that among the 9 selected mutants, 4M, 5M-B, 6M, 8M-A, and 10M maintain similar or even stronger binding affinity to hACE2 as that of the BA.1 RBD (Figure 7A). Mutants 5M-A (145.00 nM) and 8M-B (65.60 nM) have slightly weaker binding affinities to hACE2 compared to that of the BA.1 RBD. However, variants 7M and 14M have significant weaker binding affinities to hACE2. SEC analysis of the variants showed that 7M, 8M-B, and 14M are in an abnormal oligomeric state (Figure S3), which was beyond our calculation and prediction scenario. Excluding these three mutants, our *in silico* selected RBD variants achieved a Spearman’s correlation coefficient of 0.886 and a PCC of 0.726 between the predicted ΔΔG and ΔΔG derived from the BLI results (Table S11). Our predictions reported a Spearman’s correlation coefficient of 0.751 and a PCC of 0.741 between the predicted ΔΔG values and the experimental labels, on the combined multisite variants of SARS-CoV-2 RBD (from Table S7 [*n* = 13] and Table S11 [*n* = 6]).

**Figure 6:**
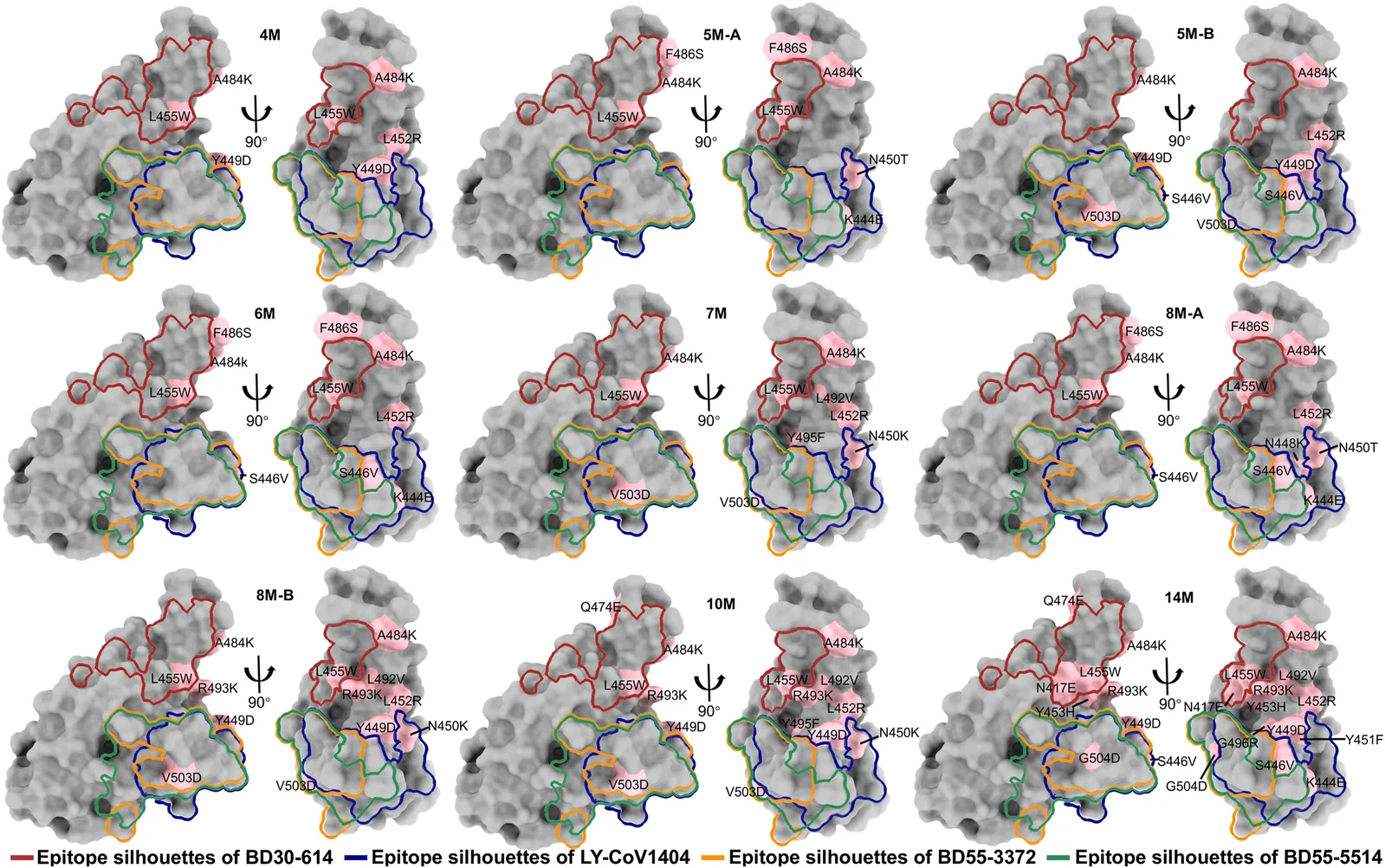
Comparisons of the mutation sites and antibody epitopes Surface-rendered representations showing mutation sites and antibody epitope regions. Mutation sites are highlighted in pink. The epitope regions of BD30-614, LY-CoV-1404, BD55-3372, and BD55-5514 are outlined with red, blue, orange, and green silhouettes, respectively. Each image displays a different RBD mutant with the corresponding epitope and mutation mapping.

**Figure 7:**
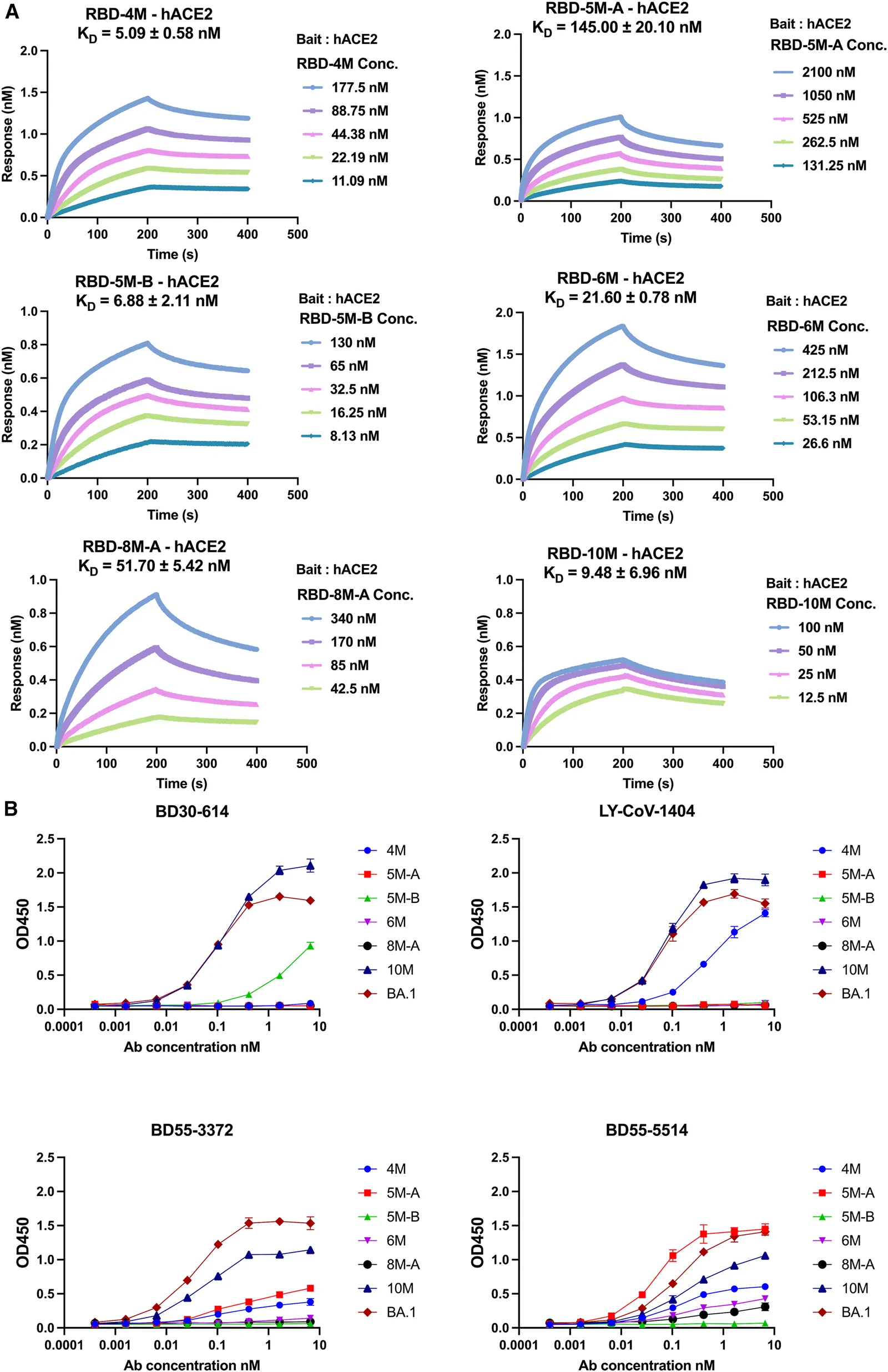
Characterization of the multisite mutation variants (A) BLI assay results showing the binding affinities of 6 selected multisite mutation variants (4M, 5M-A, 5M-B, 6M, 8M-A, and 10M) to hACE2. Each measurement was independently performed in duplicate, and the K_D_ values shown above each plot are the average along with the standard deviation (mean ± SD) of the two independent measurements. (B) Elisa assay results showing the binding of the four nAbs (BD30-614, class1; LY-CoV1404, class3; BD55-3372, class4; BD55-5514 [also known as SA55], class4) to the 6 multisite mutation variants. Antibodies were serially diluted 4-fold across 8 concentrations, and binding curves are plotted (as the average of three technical replicates with error bars represent standard deviation) on an *x* axis spaced according to the logarithmic scale of antibody concentrations, while the axis tick labels indicate the actual concentration values.

Further ELISA analysis showed that the selected variants significantly lose the binding affinity to all or some of the broadly neutralizing antibodies mentioned above (Figures 7B and S5). Among these, variants 5M-B, 6M, and 8M-A significantly lost the binding to all the four broadly neutralizing antibodies (Figure 7B). The variant 4M partially loses the binding against the LY-CoV-1404 and BD55-5514 and significantly loses the binding to others. 5M-A still keeps strong binding to BD55-5514 while significantly loses the binding to other 3 antibodies. Further analysis of the mutation sites and the epitope targeted by BD55-5514 showed that 5M-A does not contain any mutation site in the epitope of BD55-5514. Of note, the mutant 10M still keeps strong binding to BD30-614 and LY-CoV-1404, while the mutations have slightly weakened its binding to BD55-3372 and BD55-5514. The mutant 10M contains the mutation site V503D that is in the epitopes of both BD55-3372 and BD55-5514. The mutation V503D was reported to significantly affect the binding of both BD55-3372 and BD55-5514 in DMS data. According to the co-occurrence of the mutations, when V503D mutation in combined with mutations N450T/K and Y495F around the epitopes, the negative effect of V503D could be compensated somehow via a distant effect (Figure 6). Further analysis of the RBD and BD55-3372, RBD and BD55-5514 complexes showed significant attention scores on residue S446 (Table S12), suggesting that residue 446 plays a potential role in shaping the epitope of RBD antibodies BD55-3372 and BD55-5514.

To investigate the possible cause of the folding unstable variants, we evaluated our selected variants using widely used protein aggregation and solubility predictors.^73^^,^^74^^,^^75^^,^^76^^,^^77^ However, none of the models can precisely distinguish those insoluble/aggregated variants (Table 13). This indicates the limitation of current models for PPI predictions.

## Discussion

Deep learning models, especially GNNs, were already widely used in high-dimensional protein representation and prediction for protein properties.^26^^,^^78^ However, independent prediction without external predefined features still remains challenging. DGMPNN represents a comprehensive DL-style model (Figure 1), which has no more need of any pre-defined features derived through tools that use the empirical force fields. Instead, it gains knowledge on the fitness of residue identities by sensing their local environment and can be finetuned for specific predictions via an attention-based learnable GNN module. Unlike the original SAG pooling, we customized the learnable SAG pooling module by maintaining the integrity of the graph that represents the entire protein complex, without performing attention-based node pruning. In addition, the GNN module integrates node and edge features, differing from conventional self-attention mechanisms where attention is typically derived solely from node features. Our application implicates that this IPF-based architecture is readily extensible to a broad range of downstream tasks whenever task-specific labeled data are available. The framework is particularly well suited for future applications in diverse biological systems that deal with complex topological and semantic relationships among various biological entities.^79^^,^^80^ However, like other PPI prediction models, DGMPNN cannot evaluate protein folding stability. Overcoming this limitation will require future model development by incorporating additional training labels related to protein stability, solubility, and aggregation.

One distinct feature of DGMPNN is that it can distinguish those key residues at or even out of the interface of contact, indicating the capability of the model in sensing the local biochemical environment. The dataset we used for training contains only a few data points on the interaction of SARS-CoV RBD and ACE2. Our model can give acceptable prediction on the interaction of SARS-CoV2 RBD and ACE2, suggesting that the model parameters can be transferred to the complexes which shared certain homology to the case in training-set.

The current datasets assessing single-point mutation effects on protein binding affinity remain limited in scale. The entries containing multisite mutations are sparser and establishment of the related benchmark dataset still has vast area of dark-mass remained unlighted.^43^^,^^81^^,^^82^^,^^83^^,^^84^ This data scarcity may lead to over-representation of certain single point mutations and remains a major bottleneck to accurately predicting the combined effect of multisite mutations on PPI. Our tests on the prediction and evaluation of SARS-CoV-2 RBD and ACE2 interactions showed the model’s ability to predict the effect of multisite mutations, suggesting that the IPF-based models can simultaneously preserve geometric awareness of protein folding and protein-protein interfaces while capturing the local structural adaptations induced by multisite mutations. However, further validation of the model on dealing with complicated combination of mutations requires relevant benchmarks.

The IPF-based models require support from accurate protein complex structure prediction, which may be the limitation as well. Incorporating docking perturbations or conformational sampling may enhance the model performance, highlighting the need of developing physics-informed protein complex structure prediction and conformational sampling methods. These considerations emphasize the importance of integrating structural biology constraints with data-driven approaches in this domain.

### Limitations of the study

Currently, our model cannot predict essential functional attributes such as protein folding stability and aggregation propensity; incorporating these features will be crucial for the model to better serve the broader protein engineering applications. Additionally, the performance of our IPF task pre-trained model in predicting PPI mutational effects can be hindered by the reliance on potentially inaccurate structural modeling of the mutant complexes. Introducing precise structural modeling or conformational sampling methodologies for the mutant structures, may help us for achieving more precise evaluations of PPI mutational effects, especially for the multi-site variants that remodel the complex interaction interface. Furthermore, a limitation of this study is the temporal gap between the structural data used for pre-training and the Omicron-derived variants analyzed in downstream applications. The pre-training dataset was restricted to PDB structures deposited before August 2, 2021, whereas most structures of Omicron RBD variants became available subsequently. Thus, Omicron-specific structural features were not explicitly represented in the pre-training dataset. Nevertheless, DGMPNN showed reasonable performance on Omicron-derived DMS data and enabled the prediction of multisite variants, indicating that the model retains predictive performance for these variants despite the temporal gap in structural data. Future studies incorporating more recently deposited structures could further evaluate and potentially improve model performance on emerging variants.

## Resource availability

### Lead contact

Requests for further information and resources should be directed to and will be fulfilled by the lead contact, Ye Xiang (yxiang@mail.tsinghua.edu.cn).

### Materials availability

This study did not generate new unique reagents or microbial strains.

### Data and code availability

• Data reported in this paper will be shared by the lead contact upon request.
• This paper reports original custom code. The code is available from the code repository: https://github.com/MaoSihong/DGMPNN.
• Any additional information required to reanalyze the data reported in this study is available from the lead contact upon request.

## Acknowledgments

We thank the Tsinghua University Branch of the China National Center for Protein Sciences (Beijing) for providing the facility support. We thank Dr. Miao Rong and Dr. Ruofan Li for providing the materials. We thank P. F. Lai for the help with the BLI assays. This work was supported by funds from the 10.13039/501100012166National Key R&D Program of China (grant nos. 2023YFC2306300 and 2021YFA1300204), the 10.13039/501100001809National Natural Science Foundation of China (NSFC, grant nos. 31925023, 21827810, and 31861143027), the 10.13039/501100025513Beijing Frontier Research Center for Biological Structure, the SXMU-Tsinghua Collaborative Innovation Center for Frontier Medicine, and the Tsinghua-Peking Center for Life Sciences.

## Author contributions

S. Mao designed the model architecture and performed the computational case studies. S. Mao and S. Meng planned and performed the biochemical experiments. S. Mao and S. Meng analyzed data, prepared the figures, and wrote the manuscript together with Y.X. Y.X. planned and supervised all the computational and biochemical experiments, analyzed the data, and wrote the manuscript.

## Declaration of interests

The authors declare no competing interests.

## STAR★Methods

### Key resources table

**Table:** 

REAGENT or RESOURCE	SOURCE	IDENTIFIER
**Antibodies**		

horseradish-peroxidase-conjugated anti-human IgG	Promega	Cat# W4031; RRID: AB_430835

**Bacterial and virus strains**		

DH5α Competent *E.coli*	Biomed	Cat#: BC102-01

**Chemicals, peptides, and recombinant proteins**		

polyethyleneimine (PEI)	Polysciences	Cat# 24765-1
Anti-DYKDDDDK G1 Affinity Resin	GenScript	Cat# L00432-25
3×Flag peptide	QYAOBio	N/A
HEPES	Gibco	Cat# 15630080
NaCl	JSENB	Cat# JS0492; EINECS: 231-791-2
Superose 6 increase, 10/300 GL column	Cytiva	Cat# 29-0915-96
Protein A Captarose HL	HUACHUN	Cat# HB08D0701
GLYCINE	VWR LIFE SCIENCE	Cat# 0167
Tris	MLC	Cat# MG57433
SurePAGE, Bis-Tris, 10∗8, 4-20%, 15 wells	GenScript	Cat# M00657
Ni–nitrilotriacetic acid (NTA) biosensor	FortéBio	Cat# 185101
PBS	Gibco	Cat# C10010500BT
Tween 20	Beyotime	Cat# ST825
BSA	GPCSCI	Cat# AA691
3, 3' , 5, 5' -tetramethylbenzidine	Innoreagents	Cat# TMB-S-003

**Deposited data**		

DGMPNN Repo	https://github.com/MaoSihong/DGMPNN	N/A
ProteinMPNN Repo	https://github.com/dauparas/ProteinMPNN	N/A
SAGPool Repo	https://github.com/inyeoplee77/SAGPool	N/A
SKEMPI v2.0 Database	https://life.bsc.es/pid/skempi2	N/A
Protein Assemblies in the PDB (as of Aug 02, 2021)	https://www.rcsb.org	N/A
CtDock-CtCoh DMS dataset	https://www.mavedb.org/score-sets/urn:mavedb:00000077-b-2	N/A
SARS-CoV-2 RBD - hACE2 DMS dataset	github.com/jbloomlab/SARS-CoV-2-RBD_DMS_Omicron/results/final_variant_scores	N/A

**Experimental models: Cell lines**		

HEK293F	Thermo Fisher Scientific	Cat# R79007

**Recombinant DNA**		

pCDNA3.1-Strep-Flag(empty)	This Paper	N/A
pCDNA3.1-RBD-BA.1-Strep-Flag	GenBank	OM570259.1
pCDNA3.1-RBD-4M-Strep-Flag	This Paper	N/A
pCDNA3.1-RBD-5M-A-Strep-Flag	This Paper	N/A
pCDNA3.1-RBD-5M-B-Strep-Flag	This Paper	N/A
pCDNA3.1-RBD-6M-Strep-Flag	This Paper	N/A
pCDNA3.1-RBD-7M-Strep-Flag	This Paper	N/A
pCDNA3.1-RBD-8M-A-Strep-Flag	This Paper	N/A
pCDNA3.1-RBD-8M-B-Strep-Flag	This Paper	N/A
pCDNA3.1-RBD-10M-Strep-Flag	This Paper	N/A
pCDNA3.1-RBD-14M-Strep-Flag	This Paper	N/A
pCMV-10∗His-Flag(empty)	This Paper	N/A
pCMV-hACE2-10∗His-Flag	GenBank	BAB40370.1
pCMV(empty)	This Paper	N/A
pCMV_BD30-614_HC	https://opig.stats.ox.ac.uk/webapps/covabdab/	N/A
pCMV_BD30-614_LC	https://opig.stats.ox.ac.uk/webapps/covabdab/	N/A
pCMV_LY-COV1404_HC	https://opig.stats.ox.ac.uk/webapps/covabdab/	N/A
pCMV_LY-COV1404_LC	https://opig.stats.ox.ac.uk/webapps/covabdab/	N/A
pCMV_BD55-3372_HC	https://opig.stats.ox.ac.uk/webapps/covabdab/	N/A
pCMV_BD55-3372_LC	https://opig.stats.ox.ac.uk/webapps/covabdab/	N/A
pCMV_BD55-5514_HC	https://opig.stats.ox.ac.uk/webapps/covabdab/	N/A
pCMV_BD55-5514_LC	https://opig.stats.ox.ac.uk/webapps/covabdab/	N/A

**Software and algorithms**		

Octet Analysis Studio v13.0	FortéBio	N/A
GraphPad Prism v10.2.0	GraphPad	N/A
UCSF ChimeraX v1.9	https://www.cgl.ucsf.edu/chimerax/	N/A
FoldX_Linux_v4	https://foldxsuite.crg.eu/	N/A

**Other**		

ELISA plates	Corning	Cat# 9018
Octet RED384 system	FortéBio	N/A

### Experimental model and study participant details

#### Cell line and growth condition

Cell line used in this study is listed in the key resources table and was cultured under standard growth conditions (37°C, 5%CO2). HEK293F cells were obtained from Thermo Fisher Scientific (Cat# R79007) and cultured in the medium SMM 293-TII (Sino Bio-logical Inc., M293TII-1). The cell line was not further independently authenticated in our laboratory and was routinely tested for mycoplasma contamination with a negative result.

#### Bacteria strain and growth condition

E. coli strain DH5α (Biomed Cat#: BC102-01) were grown in LB broth supplemented with appropriate antibiotics at 37°C and 220 rpm. The concentrations of antibiotics used were as follows: kanamycin (Kan, 50 μg/mL); ampicillin (Amp; 100 g/mL).

### Method details

#### Featurization of protein complex

We used protein geometry information as edge embeddings in our model. Extra precomputed functional features, such as solvent-accessible surface area (SASA), evolutionary conservation, structural region classification and other physical-chemical properties, were not introduced into the model. A tensor initialized with zeros was introduced as a placeholder for node features and was involved in the subsequent message passing process. The geometric features of protein complexes were captured by encoding the Euclidean distances between atom pairs across residues. To adapt the model to the inverse protein folding pretraining task, only backbone atoms and the idealized Cβ atoms are included for the Euclidean distance calculation. For each pair of residues, distances were calculated across 25 atom pairs (Cα, C, N, O, and Cβ atom pairs) and then transformed using a Gaussian radial basis function. Specifically, 16 Gaussian kernels with centers uniformly spaced from 2 to 22 Å were applied, resulting in a 400-dimensional vector per residue to capture inter-residue geometric relationships. Relative positional embeddings for residues were introduced to the featurization of protein complexes. Sequential distances between residues were encoded using a one-hot vector of 65 bits (±32) in length. The vector encodes the relative distances between residue pairs along the peptide chain as asymmetric edge features, thereby representing the protein complex as a directed graph. To differentiate the intra-chain and inter-chain interactions within protein complexes, an additional bit is introduced to the one-hot vector, flagging whether the residues belong to the same chain or different peptide chains.

##### Model architecture

DGMPNN adopts a message-passing-neural-network^85^ (MPNN)-based encoder-decoder architecture. It consists of an encoder block, a decoder block, an attention based pooling layer, and a 2-layer MLP classifier, designed to adapt the model for downstream prediction tasks.

The encoder block consists of 3 stacked encoder layers, which take protein geometry-encoded graphs as input and generate a protein structural representation with a hidden dimension of 128 for each node and edge. A 3-layer MLP with GELU activation function was used to calculate the messages for message passing. To define the message passing region, we fetch the top-48 nearest neighbors of each center residue by ranking the Cα-Cα distances. Node features were updated using the dropout and skip connection operations, followed by layer normalization. Edge features were also updated using a 3-layer MLP with GELU activation, along with similar dropout and skip connection operations. Due to differences in the local neighborhood contexts surrounding the center residue, edge features are updated asymmetrically during message passing, consistent with the directed graph formulation. The decoder block has a similar structure as that of the encoder block, consisting of 3 stacked decoder layers with a hidden dimension of 128. The graph representation of the protein produced from the encoder was fed into the decoder to generate nodewise logits for the IPF prediction task or other node-level prediction tasks. Except for concatenating the masked protein sequence embedding to the edge features, the decoder applied a similar message passing process but did not update the edge features. Three-layer MLPs with GELU activation were used to update the nodes. Additionally, a feedforward layer using GELU activation and with the same configuration as that of the vanilla transformer, was included in both the encoder and decoder layers. The feedforward layer was placed after the node feature update step.

To adapt the model for the downstream PPI prediction task, we employed an attention based pooling layer that is modified from the Self Attention Graph Pooling strategy. In this pooling layer, an extra encoder layer was used to generate attention scores by outputting a collapsed 1-dimension value for each node (residue). This attention block is conditioned on the complex geometric representation from the encoder and the fused structure-sequence representation from the decoder to draw the node-level attention score. The SoftMax operation was applied to normalize the attention scores. To fully leverage the high dimensional structural and sequence representations captured by the encoder and decoder, updated node features from the decoder were reweighted using the attention scores by node. The reweighted node features were then summed to produce a weighted-sum of nodes features in the spatial domain as the graph-level readout, referred to a single-head attention-based graph readout. Multi-head outputs were concatenated with global max/mean pooling results, and the combined representation was fed into a subsequent MLP layer to generate the final affinity prediction for the protein complex.

#### The pretraining procedure

Protein complexes from protein assemblies in the PDB (as of Aug 02, 2021) determined by X-ray crystallography or cryo-EM were filtered according to a previously described procedure.^40^ The sequences were then clustered into 25,361 groups using a 30% sequence identity threshold. An autoregressive inverse protein folding task was performed on these protein complex data. To monitor the convergence, we measured the negative log likelihood loss between the logarithmic probability output and the one-hot encoded ground-truth sequence. During this pretraining stage, a teaching force strategy was applied. At each step of recovering the protein sequence, only part of the native sequence was visible to the model as the contextual input. The visible positions of the sequence were determined based on a randomly sampled decoding order for each sample in each epoch, while the identities of the remaining unexposed residues were kept masked. For each training epoch, a random member from each sequence cluster was selected on-the-fly to constitute the training set, referred to as the ‘query’ sequence. In protein oligomers with multiple homologous chains, a threshold of 70% sequence identity was used to identify the homologous chains to the query. All the homologous chains will be kept masked in those oligomers to prevent information leakage. The Adam optimizer (with beta1 = 0.9, beta2 = 0.98, epsilon= 10^−9^) was used to schedule the learning-rate. The models were trained with a batch size of 10,000 tokens on a single NVIDIA RTX 4090 GPU.

#### Finetune for the downstream PPI affinity prediction task

We filtered the most widely used dataset SKEMPI v2.0 by selecting the protein complexes that consist of only 2 peptide chains, yielding a dataset with 205 unique protein complexes. Furthermore, the entries denoted as no binding (n.b) or unfolding (unf) were discarded. Those denoted with in-equality binding affinity were reserved and the recorded approximate K_D_ value of the entry was used. Those unique data points (unique mutants) on each complex were kept to obtain the non-redundant entries. As the result, a subset of 2462 mutants each with a single point mutation on the 205 unique protein complexes were selected, namely S2462.

For each data point in the dataset, the backbone structure of its wild type was extracted from the SKEMPI v2.0 database. Sequence information was processed separately and fed into the model. Structural and sequence features were then integrated to obtain a comprehensive representation of each protein-protein complex, based on the weights from the pretrained model. To enable supervised downstream prediction of PPI affinity, the parameters in the pooling and readout layers were initialized from a Xavier uniform distribution. The mean squared error (MSE) loss were calculated batchwise between the model predictions and the experimental measurements (expressed as ln(K_D_)). In addition, the ΔΔG between the WT and mutant complexes was computed directly using the predicted ln(K_D_) values of the WT and mutant. Model performance and hyper-parameter selection were evaluated via a mutation-based 10-fold cross validation using a fixed partition of the dataset. Similar 10-fold cross validations were applied for the SKEMPI derived benchmarks (S1131 and S4169) in three repeats with independent data partitions from three random seeds (where the seeds include 12, 41 and 1234). To compare different model architectures, we trained each model for 500 epochs using Adam optimizer with an initial learning rate of 3 × 10^-5^. An extra linear layer as used to rescale the global graph pooling representation, ensuring compatibility with a shared MLP predictor across different model architectures. To enable a fair comparison, all finetuning models were based on the same ProteinMPNN checkpoint (namely v_48_020.pt).

#### Train on SARS-COV-2 RBD and hACE2 deep mutation scanning (DMS) data

The DMS dataset^51^^,^^52^^,^^86^ sourced from github.com/jbloomlab/SARS-CoV-2-RBD_DMS_Omicron/results/final_variant_scores is stratified by the strains of SARS-COV-2, resulting in 7 subsets. In these datasets, binding affinities (between RBD and hACE2, represented as -log_10_K_D_) had already been quantified and assigned to each single-point mutation variant. We convert the log-transformed data back to K_D_ values. The datasets were then filtered by discarding the mutants denoted as no binding. Based on the number of valid data points, the subset of SARS-COV-2 BA.1 strain baseline was selected for subsequential cross validation and analysis, including total of 3880 BA.1 RBD mutants with valid K_D_ values. The mutation-level partition is performed by randomly dividing the 20 amino acids (AAs) mutations at each site into 10 mutually exclusive sets. These subsets were used to perform 10-fold cross validation. PCC and RMSE metrics were also calculated to evaluate the model performance. An external test set, generated through experimental measurements, was also used for evaluation. This dataset consists of 33 data points, including 27 single point mutations and 6 multisite mutants, collected from previously published studies.^43^^,^^44^^,^^45^^,^^46^

#### Validation tests

To evaluate the model’s generalization ability and prevent the potential data leakage in both transfer-learning and pre-training stages, we conducted more challenging and rigorous evaluations on the SKEMPI S1131 benchmark.

For the leave-one-complex-out validation on the S1131 dataset, all mutant samples from a specific wild-type complex were held out to serve as the test set, while the model was trained on the mutant samples from all remaining complexes. The performances from former advanced models for comparison are reported under the identical benchmark settings, collected from previously published study.^32^

We conducted an experiment with sample points from complex 1R0R (*n* = 152) in the S1131 dataset as the test case to preclude the artificial performance inflation by the presence of homologous complexes during pre-training. In specific, we queried the entire pre-training database and strictly removed all protein complexes with the predicted 3D similarity score^50^ more than 0.8 or sequence identities exceeding 30% for both chains. With this homolog-purged PDB dataset, we pretrained the model with the same architecture and identical configurations. For performance comparisons, we conducted transfer-learning on S1131 with 1R0R related mutant samples excluded from both the original pretraining and the homolog-purged pretraining models. The performances are reported in mean ± SD from five independent tests with different model weight initializations.

For further validation with non-SKEMPI data, we collected a DMS dataset from the Mave database (MaveDB). This specific DMS dataset characterizes the binding affinity of *C. thermocellum* cohesin and dockerin.^87^ The corresponding reference structure is under PDB accession number 1OHZ. From the dataset, those non-synonymous mutants that contain all residues in the structure were retained, yielding a dataset (*n* = 2,609) of cohesin mutants with corresponding binding affinity for dockerin (namely CtDock-CtCoh). The stratified subset (*n* = 171), namely CtDock-CtCoh_interface and constituted by mutations of the interface residues involved in direct interactions, was selected by ChimeraX interfaces analysis tool. The ΔΔG prediction performances derived from our model tuned by the SKEMPI-S2462 dataset and the SARS-CoV2 BA.1 RBD - hACE2 DMS dataset are denoted as ‘woDMS’ and ‘wDMS’, respectively. The FoldX ΔΔG prediction performance was assessed by using the PSSM application with the default settings.

#### Selection of multisite mutation combination

To analyze effects of multisite mutations on SARS-COV-2 RBD and ACE2 interactions, we select all the interface residues of the RBD from the BA.1 strain based on the PDB structure 7WPB. Interface residues were identified using one of the following criteria: 1) any residue with at least one atom that has a van der Waals overlap (VDW overlap) greater than -0.4 Å and less than 0.6 Å from any of the ACE2 atoms, where the VDW overlap is defined as the sum of the atom-pair van der Waals radius minus the atom-pair distance; 2) any residue that has a change in buried solvent-accessible surface area (dSASA) greater than 15 Å^2^ upon complex formation. We picked 18 interface residues of the BA.1 RBD, including 449Y, 453Y, 455L, 456F, 473Y, 475A, 477N, 486F, 487N, 489Y, 493R, 494S, 496S, 498R, 500T, 501Y, 502G, 505H. Taking each site as the central site, we defined the neighboring region to explore the combination effects of multiple mutations. To make the computation achievable, we divided the RBM region into 21 patches each containing 5 residues, of which the central residue is selected from the 18 interface residues. The 21 patches cover the whole RBM region and contain 44 residues of the RBD (Table S8). The calculation was performed on a single NVIDIA RTX 4090 GPU and completed in ∼21 days, corresponding to an average runtime of less than 0.025 second per variant. In contrast, FoldX required approximately 10 seconds per variant on a single CPU core. We normalized the amino sequence frequency with the site occurrence and plotted the SeqLogo of the 44 sites. We manually picked residue sites for identifying multi-mutations that fulfill the following rules: 1) The receptor binding ability is maintained; 2) The nucleotide accessibility of each mutation defined by the NCBI standard code based on SARS-CoV-2 Reference Sequence (NCBI Reference Sequence NC_045512.2). Only single nucleotide replacement is required for the change of the amino acid residues; 3) The ability to escape antibody neutralization, as derived from statistics over different antibodies in the previous studies.^53^^,^^88^ Mutations that loses binding to most of the antibodies from the 4 epitope classes were selected.^89^

#### Gene cloning, expression and protein purification

To generate BA.1 RBD and BA.1 RBD mutants, the genes encoding BA.1 RBD (GenBank Accession: OM570259.1) and the mutants, including RBD-4M (Y449D, L452R, A484K, L455W), RBD-5M-A (N450T, K444E, A484K, L455W, F486S), RBD-5M-B (V503D, Y449D, S446V, L452R, A484K), RBD-6M (S446V, K444E, L452R, A484K, L455W, F486S), RBD-7M (L492V, Y495F, V503D, N450K, L452R, A484K, L455W), RBD-8M-A (S446V, N450T, K444E, L452R, A484K, L455W, F486S, N448K), RBD-8M-B (L492V, V503D, Y449D, R493K, N450K, L452R, A484K, L455W), RBD-10M (L492V, Y495F, V503D, Y449D, R493K, N450K, L452R, A484K, L455W, Q474E) and RBD-14M (L492V, S496R, N417E, Y449D, S446V, R493K, K444E, L452R, A484K, Y453H, G504D, L455W, Y451F, Q474E) (Table S11), were codon-optimized and synthesized (XiangHongBiotech, China). The synthesized genes were cloned to the vector pCDNA3.1 and a 1 × Strep-1 × FLAG tag was fused at the C-terminus of the RBD. The plasmids were used for transient transfection of HEK293F cells with polyethyleneimine (PEI) (Polysciences, Cat# 24765-1). The plasmid was premixed with PEI at a weight ratio of 1:3. The supernatant was harvested four days after transfection. The RBD was affinity purified from the supernatant by using anti-DYKDDDDK G1 affinity resin (GenScript, Cat# L00432-25) and eluted by using 0.1 mg/mL 3×Flag peptide (QYAOBio, China) in 20 mM HEPES at pH 7.6 and 150 mM NaCl (Figure S2A). The proteins were further purified using size-exclusion chromatography (SEC) with a Superose 6 increase, 10/300 GL column (Cytiva, Cat# 29-0915-96) in 20mM HEPES, 150mM NaCl. Peaks from SEC were collected separately and concentrated to ∼1 mg/mL. Peak fractions containing monomeric RBD and RBD mutants were used for further experiments. Mutants 7M, 8M-B and 14M did not have the monomeric peaks, so the peak fractions containing likely aggregations of the target protein were collected.

To generate human ACE2 (hACE2), the genes encoding hACE2 (GenBank Accession: BAB40370.1) was cloned to the pCMV and a 10 × His-1 × FLAG tag was introduced to the C-terminus of the recombinant protein. The plasmid was premixed with PEI at a weight ratio of 1:3 and then 6.4 mg of the plasmid-PEI mixture was used for transient transfection of 800 mL HEK293F cells at a concentration of 2 × 10^6^ cells/mL. The recombinant protein was harvested four days after transfection and was affinity purified from the supernatant by using anti-DYKDDDDK G1 affinity resin. The target protein was eluted by using 0.1 mg/mL 3×Flag peptide in 20 mM HEPES at pH 7.6 and 150 mM NaCl (Figure S2B).

Sequences of the neutralizing antibodies BD30-614, LY-COV1404, BD55-3372 and BD55-5514 were obtained from CoV-AbDab.^90^ The heavy chain and light chain genes of the antibodies were codon-optimized, synthesized and cloned into the pCMV vector. 1.6 mg of the heavy chain plasmid, 1.6 mg of the light chain plasmid and 4.8 mg PEI were mixed and were then used for the co-transfection of 800 mL HEK293F cells at a concentration of 2 × 10^6^ cells/mL. The supernatant containing antibodies was harvested 4 days after transfection. Antibody were captured by using protein A Captarose HL (HUACHUN, Cat# HB08D0701) and eluted with 0.1 M glycine at pH 3.2. The elution neutralized to pH 7.6 using 1.0 M tris at pH 9. The purified protein was analyzed by using SDS-PAGE (Figures S2C and S2D).

#### Biolayer interferometry (BLI)

RBD-ACE2 interactions were measured using an Octet RED384 system (FortéBio, Octet data acquisition v13.0) at 30 °C with an orbital shaking speed of 1000 rpm. ACE2 (0.1 mg/mL) was immobilized onto the Ni–nitrilotriacetic acid (NTA) biosensor (FortéBio, Cat# 185101) for 10 min. The biosensor was washed in PBS with 0.05% Tween 20 and 0.1% BSA for 380 s to reach a stable base state. The ACE2-loaded biosensors were incubated with RBD at different concentrations for 200 s and then immersed into PBS with 0.05% Tween 20 and 0.1% BSA for 200 s to measure the dissociation of the RBDs from the biosensor surface. BLI data was processed and analyzed by using the software Octet (version 13.0, FortéBio) and the binding mode 1:1 was selected.

#### Titration ELISA for antibody binding to RBD

The binding of RBD and RBD mutants to the antibodies were determined by titration ELISA. RBD and RBD mutants (1 μg/mL) was immobilized on ELISA plates (100 μL per well) (Corning, Cat# 9018) and incubated overnight at 4 °C. The antigen-coated plates were blocked by PBS supplemented with 3% BSA for 1 h at 37 °C. Serial dilutions of the antibodies were prepared in PBS supplemented with 3% BSA. The dilutions were added to plates and were incubated for 1 h at 37 °C. The plates were washed five times using PBS with 0.05% Tween 20 and were then incubated for 1 h at 37 °C with 100 μL per well 1:5,000 horseradish-peroxidase-conjugated anti-human IgG (Promega, Cat# W4031), which was diluted in PBS supplemented with 3% BSA. The plates were washed five times using PBS with 0.05% Tween 20 and then incubated with 100 μL of 3,3′,5,5′-tetramethylbenzidine substrate at room temperature for 150 seconds. The reaction was stopped by adding 50 μL of 1 M H_2_SO_4_. Absorbance was read at an optical wavelength of 450 nm. Data were processed and analyzed with the software Prism 10.2.0. Each experiment was repeated three times and datapoints shown in the figures are the mean values from three independent experiments.

### Quantification and statistical analysis

Statistical significance is determined using the Student’s unpaired *t* test and Steiger’s *t*-test (Steiger, J.H., 1980). The reported Pearson correlation coefficient and Spearman rank-order correlation coefficient are done Scipy.stats package. Error bars indicate standard deviation (SD). The n or N represents the number of samples in the dataset for each experiment, and is provided in figure legends, along the statistical analysis details. The meaning of asterisks is defined in figure legends or tables separately. The *p* value of ≤0.05 is considered significant.

## Supplemental information

Document S1. Figures S1–S5 and Tables S1–S13

Data S1. Source data can be found in the compressed file

Data S2. AlphaFold3 predictions of potential oligomers of multisite variants can be found in the compressed file
