# Supplementary material for: An inverse-protein-folding pre-trained model for predicting the mutational effects in protein-protein interactions
Source: iScience. 2026 Oct 6;29(10):117693. doi: 10.1016/j.isci.2026.117693 (PMC13675481; doi:10.1016/j.isci.2026.117693)
Supplement: Supplementary file 1 [file mmc1.pdf]

iScience, Volume *29*

Supplemental information

An inverse-protein-folding pre-trained model for predicting the mutational effects in protein-protein interactions

Sihong Mao, Siwei Meng, and Ye Xiang

Supplementary information

This file includes:
Supplementary Figures 1-5
Supplementary Tables 1-13

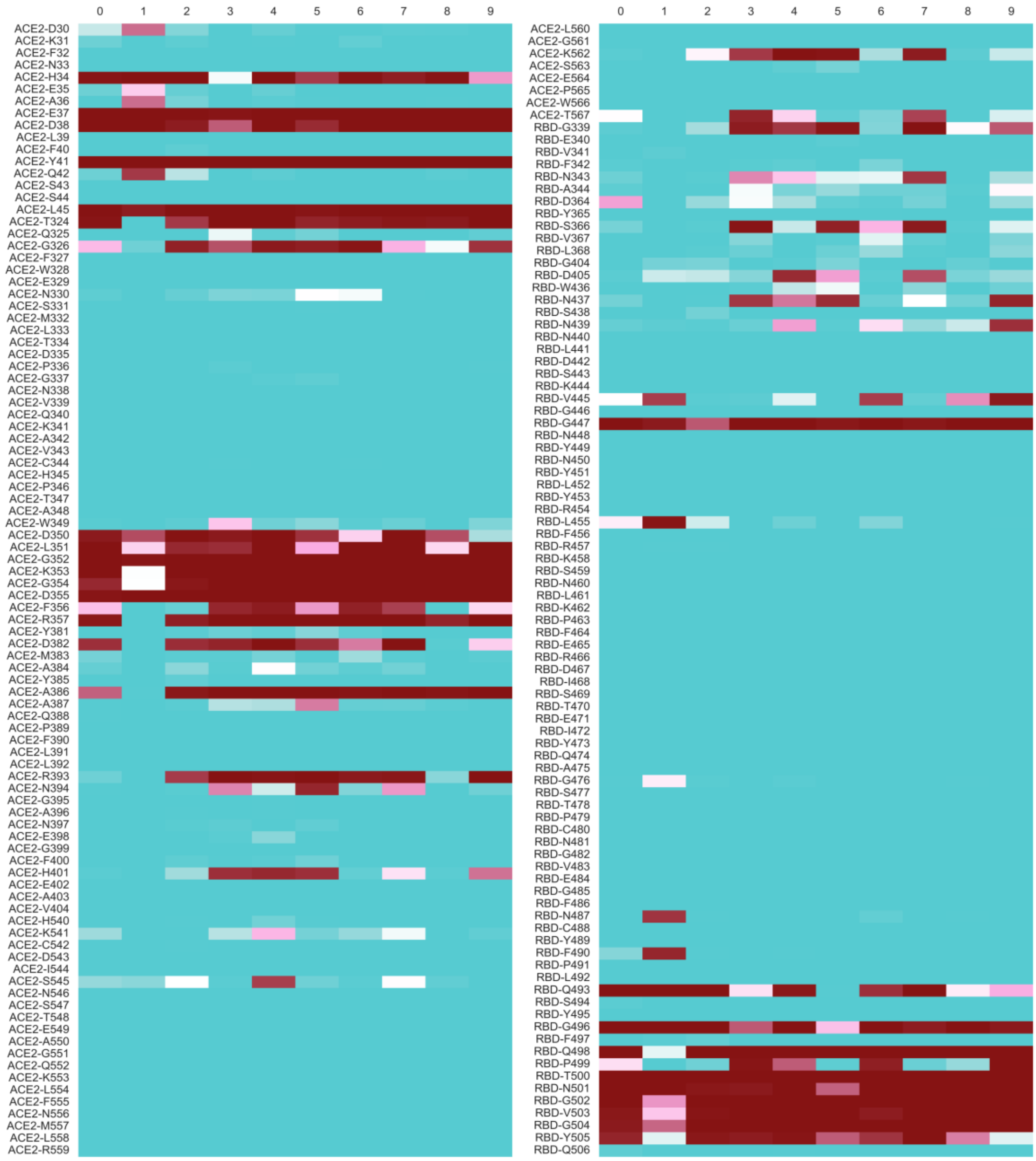

Supplementary Figure 1 Visualization of multi-head attention scores on selected residues of the RBD-hACE2 complex. Multi-head attention scores for each residue are represented as multi-cell blocks, with each cell corresponding to an individual attention head. The cells are colored according to score magnitude using a cyan to maroon color scheme. Cyan indicates low scores, while maroon indicates high score values. The number above the blocks indicate the indices of the attention heads, which share the same parameters in the model. Attention scores are shown for residues located at the interface and at identified hotspot regions.

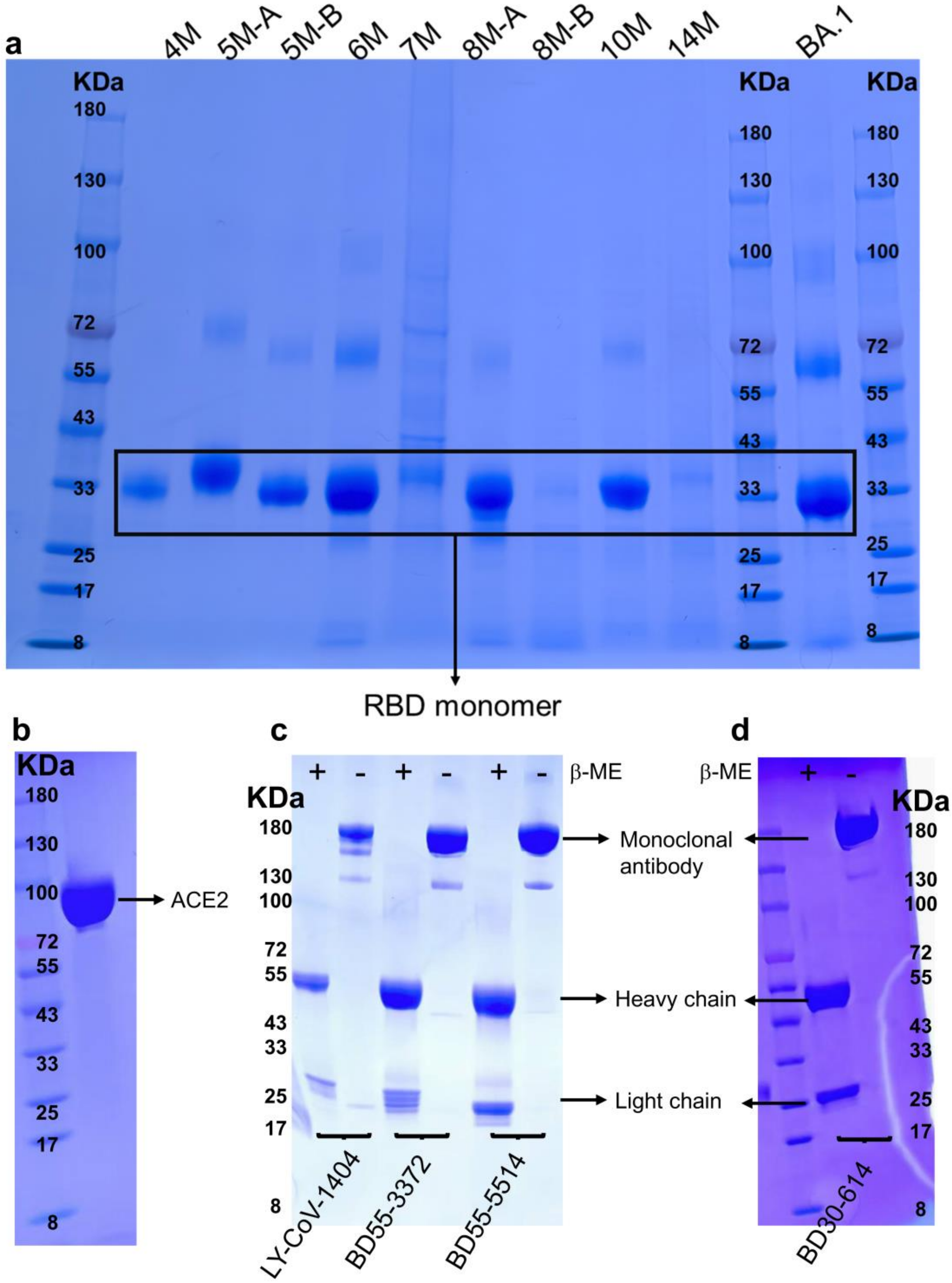

Supplementary Figure 2 SDS-PAGE analysis of the purified recombinant proteins.

(a) SDS-PAGE analysis of the purified recombinant BA.1 RBD and its mutants, including 4M, 5M-A, 5M-B, 6M, 7M, 8M-A, 8M-B, 10M, and 14M (Supplementary Table 5). (b) SDS-PAGE analysis of the purified recombinant hACE2. (c) SDS-PAGE analysis of the purified recombinant antibodies LY-CoV-1404, BD55-3372 and BD55-5514. (d) SDS-PAGE analysis of the purified recombinant antibody BD30-614.

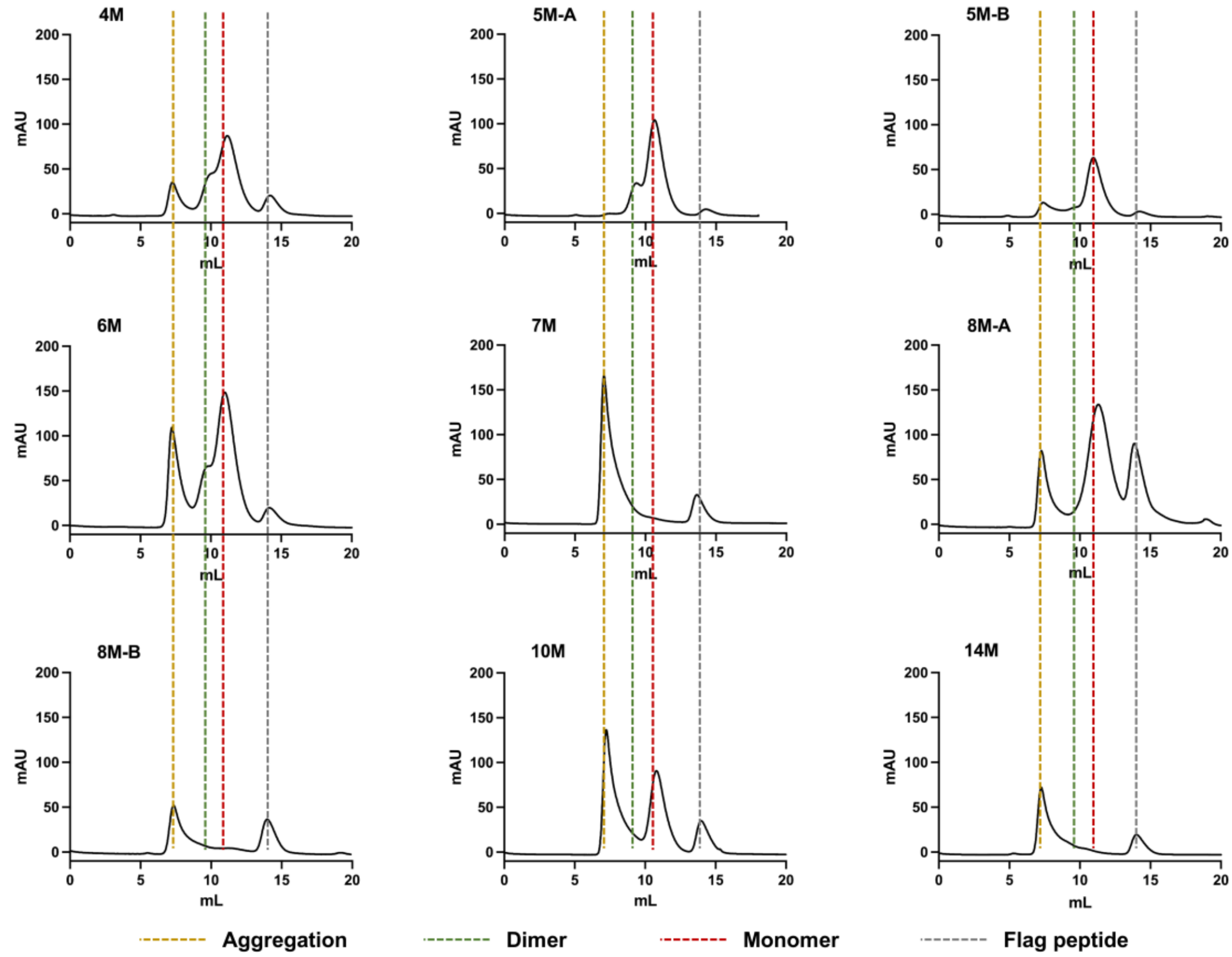

Supplementary Figure 3 SEC analysis of BA.1 RBD mutants.

The positions of the aggregated, dimeric, monomeric RBD mutant, and the flag peptide peaks are indicated by yellow, green, red, and grey lines, respectively.

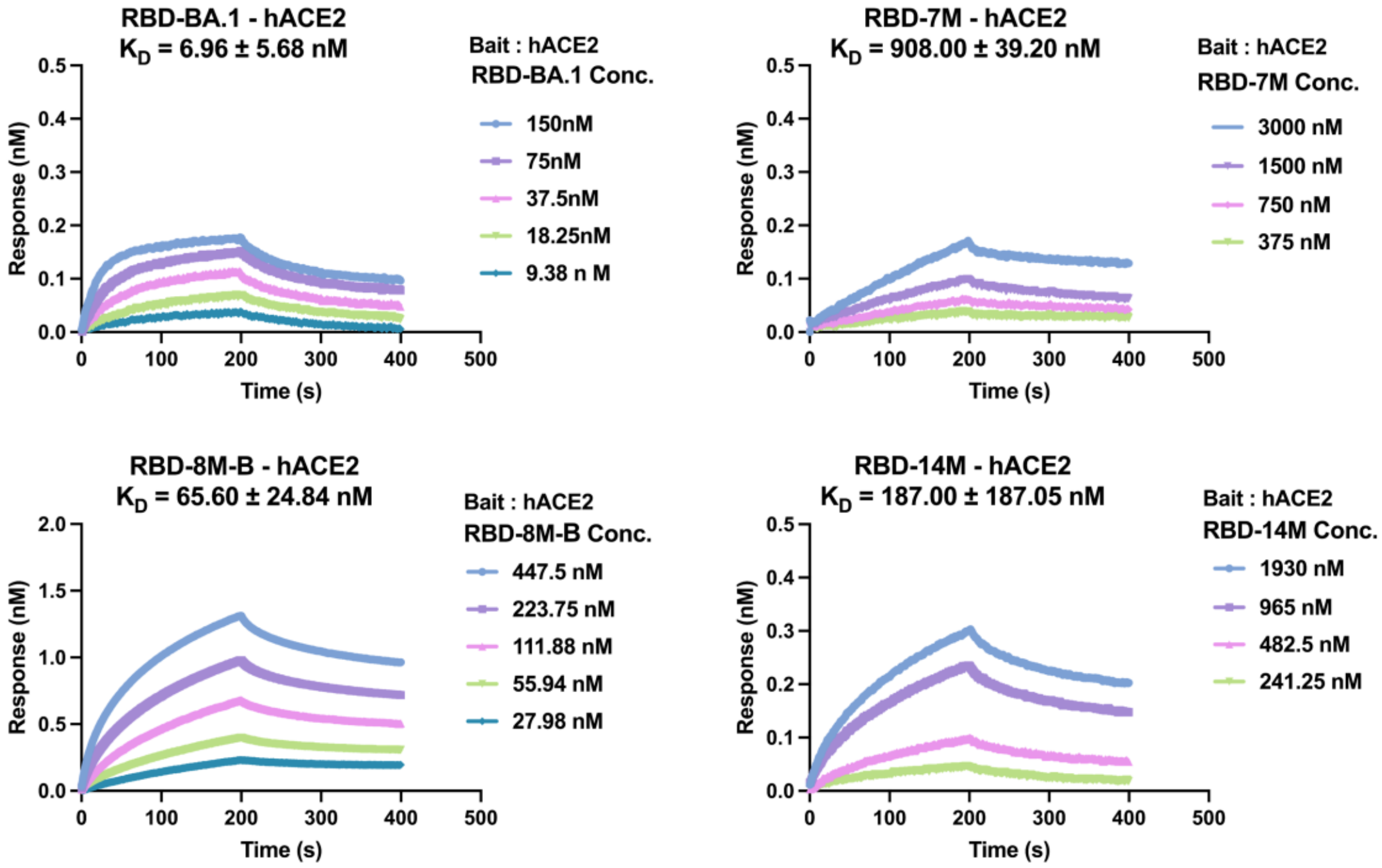

Supplementary Figure 4 BLI analysis of the binding of BA.1 RBD and its variants to hACE2.

Each measurement was independently performed in duplicate, and the K_D values shown above each plot are the average along with the standard deviation (mean ± SD) of the two independent measurements.

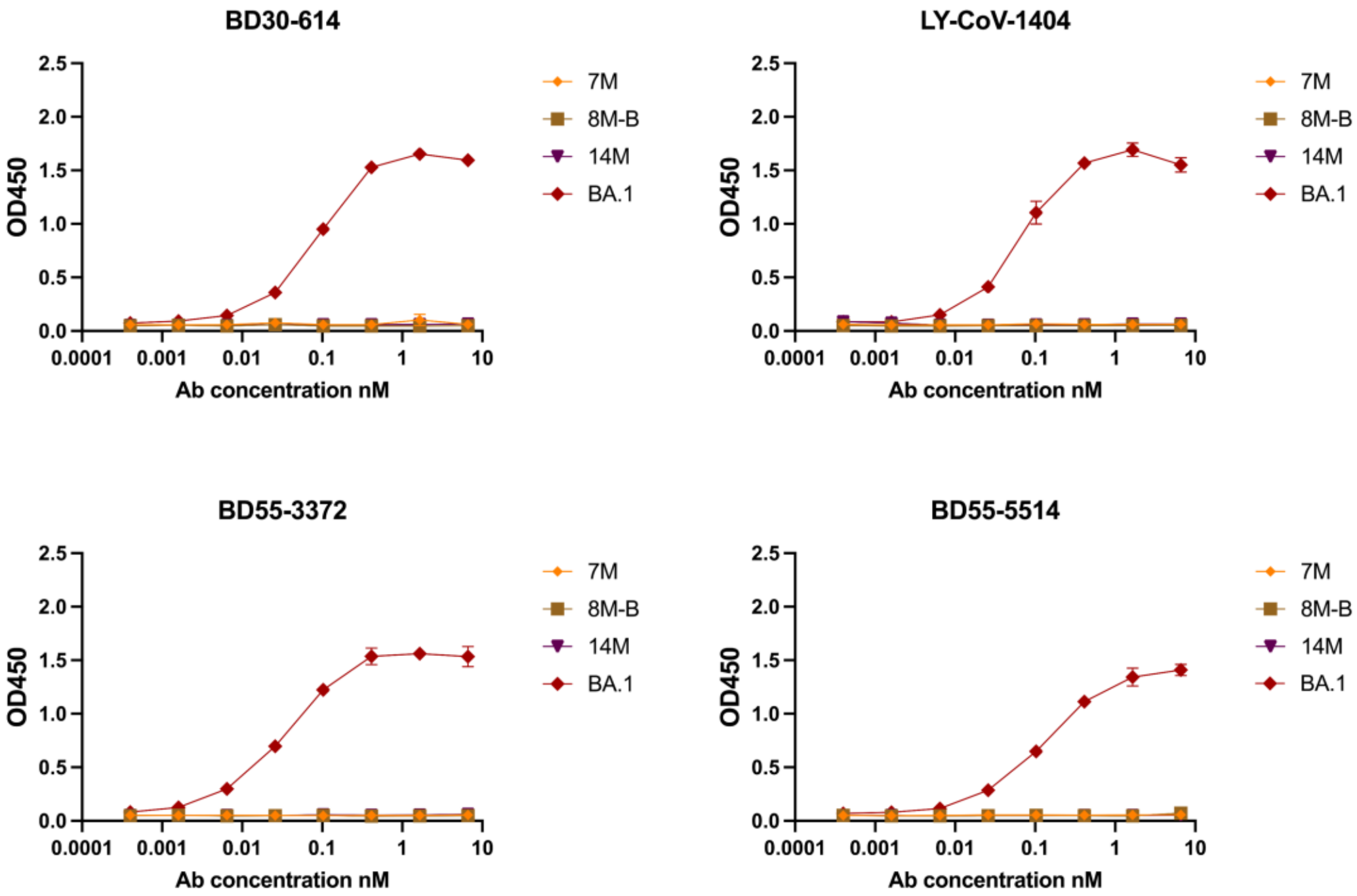

Supplementary Figure 5 Elisa analysis of the binding of BA.1 RBD variants to the four broad neutralization antibodies.

Elisa assay results showing the binding of the four nAbs (BD30-614, class1; LY-CoV1404, class3; BD55-3372, class4; BD55-5514 (also known as SA55), class4) to the three multisite variants of BA.1 RBD. RBD or RBD variants were coated onto the plates. Antibodies were serially diluted 4-fold across 8 concentrations, and binding curves are plotted (the data are presented as the mean of three technical replicates, with error bars representing the standard deviation) on an X-axis spaced according to the logarithmic scale of antibody concentrations, while the axis tick labels indicate the actual concentration values.

Supplementary Table 1 Ablation study of the model architecture.

Pooling Methods Detail			RMSE[a]	PCC[a]
Number of Attention Head	with Max Pooling	with Mean Pooling		
1	×	×	1.32	0.77
2	×	×	1.34	0.76
5	×	×	1.29	0.78
8	×	×	1.27	0.79
10	×	×	1.31	0.78
/	×	√	1.34	0.75
/	√	×	1.26	0.78
2	×	√	1.32	0.77
2	√	×	1.25	0.79
2	√	√	1.25	0.79
5	√	×	1.26	0.79
8	√	×	1.23	0.80
10	√	×	**1.22***	**0.80***

[a]The highest performance values are highlighted in bold and marked with an asterisk.

Supplementary Table 2 Performance of DGMPNN on the SKEMPI derived benchmarks.

Repeat Test[a]	Metrics	S1131	S4169
Seed_12	PCC	0.877	0.793
	RMSE	1.243	1.186
Seed_41	PCC	0.871	0.779
	RMSE	1.244	1.241
Seed_1234	PCC	0.876	0.798
	RMSE	1.223	1.173
Avg. + SD[b]	PCC	0.875±0.003	0.790±0.008

[a]The three repeated evaluations were performed using a 10-fold cross validation scheme, with each experiment using a different random seed to generate the data splits.

[b]The PCC values are expressed as mean ± SD and rounded to 3 decimal places.

Supplementary Table 3 Performance comparison of different approaches on the benchmark S1131 under the leave-one-complex-out scheme.

Method	S1131[a]	Reference
DGMPNN[b]	**0.69**	**-**
BindFormer	**0.69***	Wang et al., 2023[1]
GeoPPI	**0.58**	Liu et al., 2021[2]
TopNetTree	0.29	Wang et al., 2020[3]
FoldX	0.46	Schymkowitz, et al., 2005[4]
Flex ΔΔG	0.22	Kyle, et al., 2018[5]
MM/GBSA	0.30	Samuel, et al., 2015[6]

[a]The top 3 PCC values are highlighted in bold, with the highest value further marked with an asterisk.
[b]The DGMPNN achieves a PCC of **0.687** when rounded to three decimal places.

Supplementary Table 4 Performance comparison of the 1R0R-homolog excluded and included pretraining models.

Repeat Test[a]	Metrics	1R0R-homolog included	1R0R-homolog excluded
1	PCC	0.7160	0.6782
2	PCC	0.6693	0.6943
3	PCC	0.6710	0.6708
4	PCC	0.6867	0.7131
5	PCC	0.6803	0.6787
mean ± SD	PCC	0.6847±0.0169	0.6870±0.0151

[a]The tests were performed with five different random model weight initiations and reported in the 1R0R-complex out validation scheme with the PCC values expressed as mean ± SD.

Supplementary Table 5 Validation of DGMPNN on the deep mutation scanning dataset of *C. thermocellum* dockerin-cohesin.

Method[a]	CtDock-CtCoh[b]	CtDock-CtCoh_interface[b, c]
DGMPNN (wDMS)	**0.242**	**0.514**
DGMPNN (woDMS)	0.133	0.396
FoldX	0.145	0.460

[a]wDMS: the model was tuned by using the SKEMPI S2462 dataset and the SARS-CoV2 DMS dataset; woDMS: the model was tuned only by using the SKEMPI S2462 dataset.

[b]The best PCC values are highlighted with bold letter. The *C. thermocellum* dockerin-cohesin complex is denoted as CtDock-CtCoh in the table.

[c]Only residues at the interaction interface were included for calculation. Interface residues are defined by using the ChimeraX interface analysis tool.

Supplementary Table 6 Correlation analysis between ΔΔG and model attention.

Range	Metrics	Mean $\|\Delta\Delta G\|$[a] vs Avg. Attention[b]	Median $\|\Delta\Delta G\|$[a] vs Avg. Attention[b]
whole RBD	Pearson r	0.140	0.107
	Spearman ρ	0.221	0.241
RBM only (438-506)	Pearson r	0.189	0.145
	Spearman ρ	**0.508**	**0.504**
non-RBM	Pearson r	-0.088	-0.084
	Spearman ρ	0.002	0.049

[a]The average/median of the absolute ΔΔG value over 20 amino acid residue types from each site is used for correlation analysis.

[b]The average of attention score over all attention heads of each site is used for correlation analysis.

Supplementary Table 7 Comparison of model predictions with experimental ΔΔG values for multi-site mutation variants of SARS-CoV2.

SARS-CoV2 Variant	Mutations	Predicted ΔΔG (kcal/mol) [a, b]	Experimental ΔΔG (kcal/mol) [a, b, c]	Summation of DMS ΔΔG (kcal/mol) [b, c]
-	K417N, E484K	0.005	0.711	0.734
-	K417T, E484K	-0.002	0.427	0.550
Theta (P.3)	E484K, N501Y	-2.719	-1.590	-0.644
Beta (B.1.351)	K417N, E484K, N501Y	-2.715	-0.778	-0.021
Gamma (P.1)	K417T, E484K, N501Y	-2.729	-1.010	-0.105
-	Q498R, N501Y	-4.454	-1.170	-0.585
Delta (B.1.617.2)	L452R, T478K	0.011	-0.297	-0.296
Kappa (B.1.617.1)	L452R, E484Q	0.004	0.211	-0.177
Lambda (C.37)	L452Q, F490S	-0.012	-1.387	-0.349
Mu (B.1.621)	R346K, E484K, N501Y	-2.736	-0.519	-1.849
Omicron (BA.2.75)	G339H, S371F, S373P, S375F, T376A, D405N, R408S, K417N, N440K, G446S, N460K, S477N, T478K, E484A, Q498R, N501Y, Y505H	-4.671	-1.362	3.867
Omicron (BA.4/5)	G339D, S371F, S373P, S375F, T376A, D405N, R408S, K417N, N440K, L452R, S477N, T478K, E484A, F486V, Q498R, N501Y, Y505H	-4.005	-0.248	2.969
Omicron (XBB)	G339H, R346T, L368I, S371F, S373P, S375F, T376A, D405N, R408S, K417N, N440K, V445P, G446S, N460K, S477N, T478K, E484A, F486S, F490S, Q498R, N501Y, Y505H	-3.388	-0.567	4.712
BA.1 + R498C	G339D, S371L, S373P, S375F, K417N, N440K, G446S, S477N, T478K, E484A, Q493R, G496S, Q498C, N501Y, Y505H	3.233	4.423	4.423
BA.1 + Y501A	G339D, S371L, S373P, S375F, K417N, N440K, G446S, S477N, T478K, E484A, Q493R, G496S, Q498R, N501A, Y505H	4.453	3.934	3.934

[a]The calculated Pearson's correlation between the two columns is 0.565 (N=13). The two mutants (in red) with disruptive mutations were used as negative controls and excluded from the correlation calculation.

[b]Predicted ΔΔG values were calculated via the formula $\Delta\Delta G = \Delta G_{mut} - \Delta G_{wt} = 0.001 * R * T * ln(K_{D_{mut}} / K_{D_{wt}})$, where R (universal gas constant) takes the value of 1.9872036 ($kcal \cdot K^{-1} \cdot mol^{-1}$), T (absolute temperature) takes the value of 298.15 K.

[c]The calculated Pearson's correlation between the two columns is 0.091 (N=13). The two mutants (in red) with disruptive mutations were used as negative controls and excluded from the correlation calculation.

Supplementary Table 8 Information of the patches used for saturated mutation screening.

Center Residue	Selected Sites
449	447, 448, 449, 496, 498
449	449, 450, 451, 452, 494
453	452, 453, 454, 492, 493
455	417, 421, 454, 455, 456
456	454, 455, 456, 457, 491
473	472, 473, 474, 475, 489
475	473, 474, 475, 476, 487
477	474, 476, 477, 478, 479
486	475, 476, 485, 486, 487
487	484, 485, 486, 487, 489
489	456, 473, 475, 487, 489
493	452, 453, 492, 493, 494
494	449, 451, 493, 494, 495
496	495, 496, 497, 498, 501
498	444, 446, 497, 498, 499
498	448, 450, 451, 452, 498
500	498, 499, 500, 501, 502
501	496, 498, 501, 502, 505
501	497, 498, 499, 500, 501
502	501, 502, 503, 505, 506
505	497, 503, 504, 505, 506

Supplementary Table 9 Information on RBD mutations used for in-silicon multi-site mutation screening.

Site Index	Native AA	Selected Mutable AA	Site Index	Native AA	Selected Mutable AA
417	N	N, E	479	P	P, L
421	Y	Y, N	484	A	A, K, V
444	K	K, E	485	G	G, R
446	S	S, V	486	F	F, S
447	G	G, D, P	487	N	N
448	N	N, K	489	Y	Y, H
449	Y	Y, D	491	P	P
450	N	N, T, K	492	L	L, V
451	Y	Y, F	493	R	R, K
452	L	L, R	494	S	S, P
453	Y	Y, H	495	Y	F, H
454	R	R	496	S	S, R
455	L	L, W	497	F	F
456	F	F, I, T	498	R	R
457	R	R	499	P	P
472	I	I, F	500	T	T
473	Y	Y, S	501	Y	Y
474	Q	Q, E	502	G	G
475	A	A, V	503	V	V, D
476	G	G, S	504	G	G, D
477	N	N, G, I	505	H	H
478	K	K, P	506	Q	Q

Supplementary Table 10 Information on the multi-site variants used for in-vitro validation.

Mutant ID[a]	Mutation(s)	Mutation(s) Count	Predicted $\ln K_D$[b]
4M	Y449D, L452R, A484K, L455W	4	-22.7974
5M-A	N450T, K444E, A484K, L455W, F486S	5	-19.0849
5M-B	V503D, Y449D, S446V, L452R, A484K	5	-19.7094
6M	S446V, K444E, L452R, A484K, L455W, F486S	6	-19.4516
**7M**	L492V, Y495F, V503D, N450K, L452R, A484K, L455W	7	-22.1214
8M-A	S446V, N450T, K444E, L452R, A484K, L455W, F486S, N448K	8	-18.6698
**8M-B**	L492V, V503D, Y449D, R493K, N450K, L452R, A484K, L455W	8	-22.7460
10M	L492V, Y495F, V503D, Y449D, R493K, N450K, L452R, A484K, L455W, Q474E	10	-22.3548
**14M**	L492V, S496R, N417E, Y449D, S446V, R493K, K444E, L452R, A484K, Y453H, G504D, L455W, Y451F, Q474E	14	-19.0839

[a]RBD variants in soluble aggregations are marked in bold red.

[b]The predicted $\ln K_D$ is the direct output of the model prediction.

Supplementary Table 11 Comparison of model predictions with experimental ΔΔG values for multi-site mutation mutants.

Mutant ID[a]	Mutations	predicted ΔΔG (kcal/mol)[b, c]	experimental ΔΔG (kcal/mol)[b, c]
4M	Y449D, L452R, A484K, L455W	-0.90	-0.19
5M-A	N450T, K444E, A484K, L455W, F486S	1.29	1.80
5M-B	V503D, Y449D, S446V, L452R, A484K	0.93	-0.01
6M	S446V, K444E, L452R, A484K, L455W, F486S	1.08	0.67
7M	L492V, Y495F, V503D, N450K, L452R, A484K, L455W	-0.50	2.89
8M-A	S446V, N450T, K444E, L452R, A484K, L455W, F486S, N448K	1.54	1.19
8M-B	L492V, V503D, Y449D, R493K, N450K, L452R, A484K, L455W	-0.87	1.33
10M	L492V, Y495F, V503D, Y449D, R493K, N450K, L452R, A484K, L455W, Q474E	-0.64	0.18
14M	L492V, S496R, N417E, Y449D, S446V, R493K, K444E, L452R, A484K, Y453H, G504D, L455W, Y451F, Q474E	1.30	1.95

[a]RBD mutants showing only an aggregation peak are in bold red.

[b]The calculated Spearman's correlation and Pearson's correlation between the two columns are 0.886 (N=6, excluding 7M, 8M-B and 14M) and 0.726 (N=6, excluding 7M, 8M-B and 14M), respectively.

[c]Predicted ΔΔG values were calculated via the formula $\Delta\Delta G = \Delta G_{mut} - \Delta G_{wt} = 0.001 * R * T * ln(K_{D_{mut}} / K_{D_{wt}})$, where R (universal gas constant) takes the value of 1.9872036 ($kcal \cdot K^{-1} \cdot mol^{-1}$), T (absolute temperature) takes the value of 298.15.

Supplementary Table 12 Attention scores on residues surrounding S446 in the complexes with BD55-3372 and BD55-5514.

Site on RBD	Attention Score[a]		Site on RBD	Attention Score[a]	
	BD55-3372	BD55-5514		BD55-3372	BD55-5514
439	0.2260923	0.6202392	473	0.0000304	0.0000233
440	0.1174946	0.9972401	474	0.0000601	0.0000446
441	0.2444418	0.5486973	475	0.0000885	0.0000409
442	0.0000950	0.0001052	476	0.0001951	0.0000573
443	0.3202638	0.2884833	477	0.0105914	0.0005918
444	0.0031205	0.0002806	478	0.0213388	0.0013816
445	0.9772944	0.9985829	479	0.0165387	0.0031850
446	0.8998259	0.6192629	480	0.1688158	0.1637069
447	0.9849124	0.4063206	481	0.1262067	0.0183999
448	0.0002282	0.0002714	482	0.0003625	0.0001494
449	0.0001371	0.0002010	483	0.0001413	0.0000634
450	0.0286071	0.4310733	484	0.0375326	0.0168407
451	0.0000331	0.0000352	485	0.0001436	0.0002053
452	0.0000242	0.0000258	486	0.0135450	0.0019300
453	0.0000396	0.0000332	487	0.0411969	0.0041180
454	0.0000945	0.0000844	488	0.0002989	0.0002301
455	0.0000411	0.0000582	489	0.0001893	0.0001206
456	0.0000238	0.0000196	490	0.4966853	0.4886904
457	0.0000209	0.0000208	491	0.0000232	0.0000205
458	0.0000229	0.0000191	492	0.0000385	0.0000316
459	0.0000168	0.0000170	493	0.0000306	0.0000243
460	0.0000201	0.0000203	494	0.0000338	0.0000331
461	0.0000204	0.0000206	495	0.0705599	0.0010867
462	0.0000198	0.0000205	496	0.6520185	0.9082536
463	0.0000241	0.0000242	497	0.0996694	0.0754217
464	0.0000245	0.0000245	498	0.9952743	0.8734079
465	0.0000220	0.0000206	499	0.9551985	0.9608547
466	0.0000700	0.0000718	500	0.9921735	0.8627955
467	0.0000189	0.0000189	501	0.9502711	0.9992191
468	0.0000208	0.0000206	502	0.9608723	0.9936487
469	0.0000176	0.0000174	503	0.9968055	0.9993764
470	0.0000453	0.0000332	504	0.9881067	0.7299147
471	0.0000346	0.0000254	505	0.7579489	0.9688445
472	0.0000741	0.0000554	506	0.9982167	0.7169395

[a]The attention score on RBM in the complex with BD55-3372 and BD55-5514 is extracted from the DGMPNN prediction with the structural models 7WRO (PDB accession number) and 7Y0W (PDB accession number), respectively. The RBD chain in the complex is superimposed and substituted by BA.1 RBD and all the non-relevant chains are removed.

Supplementary Table 13 Aggregation prediction of the multi-site variants using different tools.

Name[a]	CamSol[b]	Protein-Sol[b]	SOLUPROT[b]	AGGRESCAN[b]	NetSolP[b]
4M	-0.241	0.542	0.693	4.00	0.567
5M-A	-0.335	0.463	0.705	6.20	0.536
5M-B	-0.214	0.526	0.692	3.40	0.585
6M	-0.262	0.491	0.681	5.20	0.537
**7M**	-0.329	0.587	0.650	4.40	**0.571**
8M-A	-0.299	0.519	0.675	6.00	0.531
**8M-B**	-0.257	**0.603**	0.669	**2.80**	0.533
10M	-0.276	0.574	0.670	3.00	0.552
**14M**	**-0.197**	0.429	**0.705**	2.90	0.542
Classification metrics[c]	threshold = -0.19<br>recall = 1<br>precision = 1/3<br>FP/(TP+FP) = 2/3<br>accuracy = 1/3	threshold = 0.61<br>recall = 1<br>precision = 1/3<br>FP/(TP+FP) = 2/3<br>accuracy = 1/3	threshold = 0.71<br>recall = 1<br>precision = 1/3<br>FP/(TP+FP) = 2/3<br>accuracy = 1/3	threshold = 2.8<br>recall = 1<br>precision = 1/3<br>FP/(TP+FP) = 2/3<br>accuracy = 1/3	threshold = 0.58<br>recall = 1<br>precision = 3/8<br>FP/(TP+FP) = 5/8<br>accuracy = 4/9

[a]RBD variants in the soluble aggregation form are in **bold red**.

[b]Tools used for predicting the protein solubility or aggregation tendency[7–11]. For tools highlighted with a green background, higher scores indicate greater protein solubility; For tools highlighted with a yellow background, higher scores indicate a greater tendency for protein aggregation. Threshold values for each tool are shown in **bold dark red**.

[c]The threshold for each tool is set to ensure a 100% recall rate, such that every experimentally confirmed aggregated variant was predicted as insoluble/aggregation-prone at the selected threshold. The precision (TP/(TP+FP)), accuracy((TP+TN)/(TP+FP+TN+FN)), and the FP/(TP+FP) were called with the threshold setting.

References

1. Wang, G. *et al.* Deep-learning-enabled protein–protein interaction analysis for prediction of SARS-CoV-2 infectivity and variant evolution. *Nat Med* **29**, 2007–2018 (2023).
2. Liu, X., Luo, Y., Li, P., Song, S. & Peng, J. Deep geometric representations for modeling effects of mutations on protein-protein binding affinity. *PLOS Computational Biology* **17**, e1009284 (2021).
3. Wang, M., Cang, Z. & Wei, G.-W. A topology-based network tree for the prediction of protein–protein binding affinity changes following mutation. *Nat Mach Intell* **2**, 116–123 (2020).
4. Schymkowitz, J. *et al.* The FoldX web server: an online force field. *Nucleic Acids Research* **33**, W382–W388 (2005).
5. Barlow, K. A. *et al.* Flex ddG: Rosetta Ensemble-Based Estimation of Changes in Protein–Protein Binding Affinity upon Mutation. *J. Phys. Chem. B* **122**, 5389–5399 (2018).
6. Genheden, S. & Ryde, U. The MM/PBSA and MM/GBSA methods to estimate ligand-binding affinities. *Expert Opinion on Drug Discovery* **10**, 449–461 (2015).
7. Sormanni, P., Aprile, F. A. & Vendruscolo, M. The CamSol method of rational design of protein mutants with enhanced solubility. *J Mol Biol* **427**, 478–490 (2015).
8. Hebditch, M., Carballo-Amador, M. A., Charonis, S., Curtis, R. & Warwicker, J. Protein-Sol: a web tool for predicting protein solubility from sequence. *Bioinformatics* **33**, 3098–3100 (2017).
9. Hon, J. *et al.* SoluProt: prediction of soluble protein expression in Escherichia coli. *Bioinformatics* **37**, 23–28 (2021).
10. Conchillo-Solé, O. *et al.* AGGRESCAN: a server for the prediction and evaluation of 'hot spots' of aggregation in polypeptides. *BMC Bioinformatics* **8**, 65 (2007).
11. Thumuluri, V. *et al.* NetSolP: predicting protein solubility in Escherichia coli using language models. *Bioinformatics* **38**, 941–946 (2022).
